# Functional Equivalence and Conserved Sexual Dimorphism in the Gut Microbiome: A Cross-Species Meta-analysis

**DOI:** 10.1007/s00239-026-10338-z

**Published:** 2026-07-29

**Authors:** Jorge Luis Gutiérrez-Ávila, Gabriel Alfonso Gutiérrez-Rebolledo, Rodolfo Gamaliel Avila-Bonilla, María Elena Sánchez Pardo

**Affiliations:** 1 Instituto Politécnico Nacional, Escuela Nacional de Ciencias Biológicas—Campus Zacatenco, Unidad Profesional Adolfo López Mateos, Zacatenco, Av. Wilfrido Massieu 399, Colonia Nueva Industrial Vallejo, C.P., Alcaldía Gustavo A. Madero, Mexico City, 07738 Mexico; 2 Departamento de Genética y Biología Molecular, Centro de Investigación y de Estudios Avanzados del Instituto Politécnico Nacional, Av. IPN 2508, Mexico City, Mexico

**Keywords:** Gut microbiome, Functional equivalence, Sexual dimorphism, Ecological filtering, Metagenomics

## Abstract

The murine model is a standard system in translational microbiome research, yet its functional equivalence to the human microbiome remains debated. To evaluate its translational validity, we conducted a comparative whole-genome shotgun (WGS) metagenomic meta-analysis, integrating an initial retrieval of 520 datasets from 5 independent cohorts (BioProjects) across *Homo sapiens *(*n* = 202), *Mus musculus *(*n* = 75), and *Drosophila melanogaster *(*n* = 243) samples. Taxonomic and functional profiles were evaluated using strict bioinformatic quality control and batch-effect mitigation. Taxonomic profiling revealed pronounced divergence driven by host-specific ecological constraints and filtering. However, metabolic reconstruction demonstrated substantial functional equivalence, supporting the functional redundancy hypothesis for core mammalian metabolic circuits. We also noted a methodological vulnerability in our dataset: a low-depth murine sample clustered with invertebrate profiles, suggesting that technical noise or insufficient depth might artificially compress mammalian functional diversity. Comparative analysis identified sex-biased metabolic pathways conserved across mammalian hosts. Specifically, we observed a consistent enrichment of steroid metabolism in females and mineralocorticoid regulation in males. These findings indicate that functional conservation between humans and mice is modular rather than global. Consequently, the translational value of the murine model lies in domain-specific functional equivalence rather than taxonomic imitation. Moreover, the conservation of sex-specific metabolic signatures suggests that biological sex is a fundamental organising principle of microbiome function. This study highlights the necessity of mapping conserved metabolic modules and rigorously controlling inter-study variance to effectively deploy murine models in biomedical research.

**Supplementary Information:**

The online version contains supplementary material available at https://doi.org/10.1007/s00239-026-10338-z.

## Introduction

*Mus musculus* has long been considered the principal model organism for translational biomedical research due to its genetic tractability, controlled environmental reproducibility, and physiological homology with humans (Perlman 2016; Rosenthal and Brown 2007). In microbiome research, murine models have been extensively employed to investigate host–microbe interactions, metabolic regulation, immune modulation, and disease mechanisms (Ley et al. 2005; Turnbaugh et al. 2007). However, while the advent of Whole-Genome Sequencing (WGS) metagenomics has revolutionised our ability to reconstruct the functional potential of these communities (Mancabelli et al. 2017), a critical bottleneck remains: the “translational gap.” Most mechanistic insights are derived from the murine model based on the premise of evolutionary conservation, yet the validity of this extrapolation is increasingly scrutinised (McGee and Huttenhower 2021).

Despite sharing a common mammalian ancestry and approximately 85% of genomic coding sequences(Waterston et al. 2002), humans and mice exhibit fundamental anatomical and physiological dissimilarities that impose distinct selective pressures on their microbial inhabitants. The murine gastrointestinal tract is characterised by a non-glandular forestomach (pH 3–4), a disproportionately large cecum dedicated to fermentation, and a metabolic rate sevenfold higher than that of humans(Hugenholtz and de Vos 2017). These physiological divergences raise a fundamental question: Does the murine microbiome functionally recapitulate the human ecosystem, or is it a distinct entity shaped by disparate environmental niches?

Comparative analyses have demonstrated that while broad phylum-level similarities exist between humans and mice, taxonomic overlap at lower hierarchical levels is limited, and strain-level differences are pronounced (Krych et al. 2013; Xiao et al. 2015). Functional redundancy is used to explain this phenomenon, whereby distinct taxa encode analogous metabolic capabilities (Turnbaugh et al. 2007).

Nevertheless, whether such functional equivalence genuinely preserves metabolic architecture across host species remains incompletely resolved. Consequently, inferring human physiological mechanisms from murine data requires caution, as ecological divergence driven by host-specific pressures may lead to substantial discrepancies in functional capacity (Nguyen et al. 2015).

Although the influence of biological sex on the microbiome is well established—with recent evidence identifying genetic strategies for the sex-biased persistence of specific gut microbes across the human lifespan (Tarracchini et al. 2023)—its evolutionary conservation across disparate mammalian species remains poorly understood. Although sexual dimorphism is well-documented in murine physiology, where it significantly influences immune responses and metabolic regulation (Markle et al. 2013; Org et al. 2016), it remains unclear whether sex-biased microbial functions represent evolutionarily conserved traits or species-specific phenomena (McGee and Huttenhower 2021). Determining this conservation is a critical requirement for validating the translational utility of murine models in sex-stratified biomedical research.

Given these unresolved issues, systematic cross-species functional comparisons are required to determine whether murine microbiomes faithfully reproduce human metabolic architectures at the pathway level. In this study, we evaluate the translational validity of the murine model through a comprehensive metagenomic meta-analysis. To provide a robust evolutionary framework, we include *Drosophila melanogaster*. This organism has emerged as a powerful model for dissecting fundamental principles of gut microbiome ecology, such as community assembly, niche construction, and host-microbe metabolic signalling (Ludington and Ja 2020). Indeed, previous studies have utilized the fly to model conserved pathways relevant to mammalian health, including the modulation of host growth and insulin signaling by *Acetobacter* species (Shin et al. 2011), suggesting a degree of functional conservation across vast evolutionary distances. By incorporating this invertebrate as an outgroup—a departure from traditional pairwise comparisons—we can rigorously distinguish conserved ‘mammalian traits’ (shared by mice and humans but absent in flies) from broader, kingdom-level symbiotic patterns.

## Methodology

### Study Design and Data Acquisition

To ensure sufficient statistical power and representativeness, the final curated dataset consisted of 520 datasets of biological samples distributed across the three-host species. The demographic breakdown by biological sex was as follows: *Homo sapiens* comprised 202 samples (136 females, 66 males); *Mus musculus* comprised 75 samples (39 females, 36 males); and *Drosophila melanogaster* 243 samples (18 females, 225 males) Full metadata, including accession numbers and sequencing depth per sample, are detailed in Supplementary Table S1.

A comparative metagenomic meta-analysis was conducted using public Whole Genome Shotgun (WGS) sequencing data deposited in the Sequence Read Archive (SRA, https://www.ncbi.nlm.nih.gov/sra/) of the National Centre for Biotechnology Information (NCBI, https://www.ncbi.nlm.nih.gov/). Representative gut microbiome studies corresponding to three model organisms were selected: *Homo sapiens*, *Mus musculus*, and *Drosophila melanogaster*. Metadata associated with each BioProject were manually curated to homogenise variables of interest, including sex (Male/Female), organism, and experimental group. Comprehensive metadata for all included datasets is available in the Supplementary Information (Supplementary Table S1). Samples exhibiting incomplete metadata or insufficient sequencing depth (< 1 million reads) were excluded from the analysis. While this threshold aligns with accepted literature standards for robust community-level profiling (Hillmann et al. 2018), its implementation was empirically mandated in our dataset to prevent sparsity-induced technical dropouts, which artefactually compressed mammalian functional diversity. A detailed rationale is provided in the Supplementary Methods (Sect.  1.4). Following stringent quality control and host DNA depletion, a total of 520 high-quality independent whole-genome sequencing (WGS) gut microbiome samples were retained for downstream analysis, comprising *H. sapiens* (*n* = 202), *M. musculus* (*n* = 75), and *D. melanogaster* (*n* = 243). A detailed of computational depletion of host DNA is provided in the Supplementary Methods. Comprehensive sample metadata, including BioProject accessions and individual sequencing metrics, are detailed in Supplementary Table S1.

### Sampling Effort and Taxonomic Saturation

To evaluate whether the sampling effort provided sufficient power to represent the ecosystem backbone, we performed a taxonomic saturation analysis using species accumulation curves (rarefaction). This approach determines the relationship between the cumulative number of samples analysed and the observed taxonomic richness (Gotelli and Colwell 2001). Sampling adequacy was numerically quantified by calculating the slope of the rarefaction curve at the final sampling point (*n* = 75 for *M. musculus*). A slope approaching zero indicates that the core community has been successfully captured and that further sampling would yield diminishing returns in terms of new taxonomic discoveries (Colwell et al. 2004).

### Bioinformatic Pipeline and Data Pre-processing

**Fig. 1:**
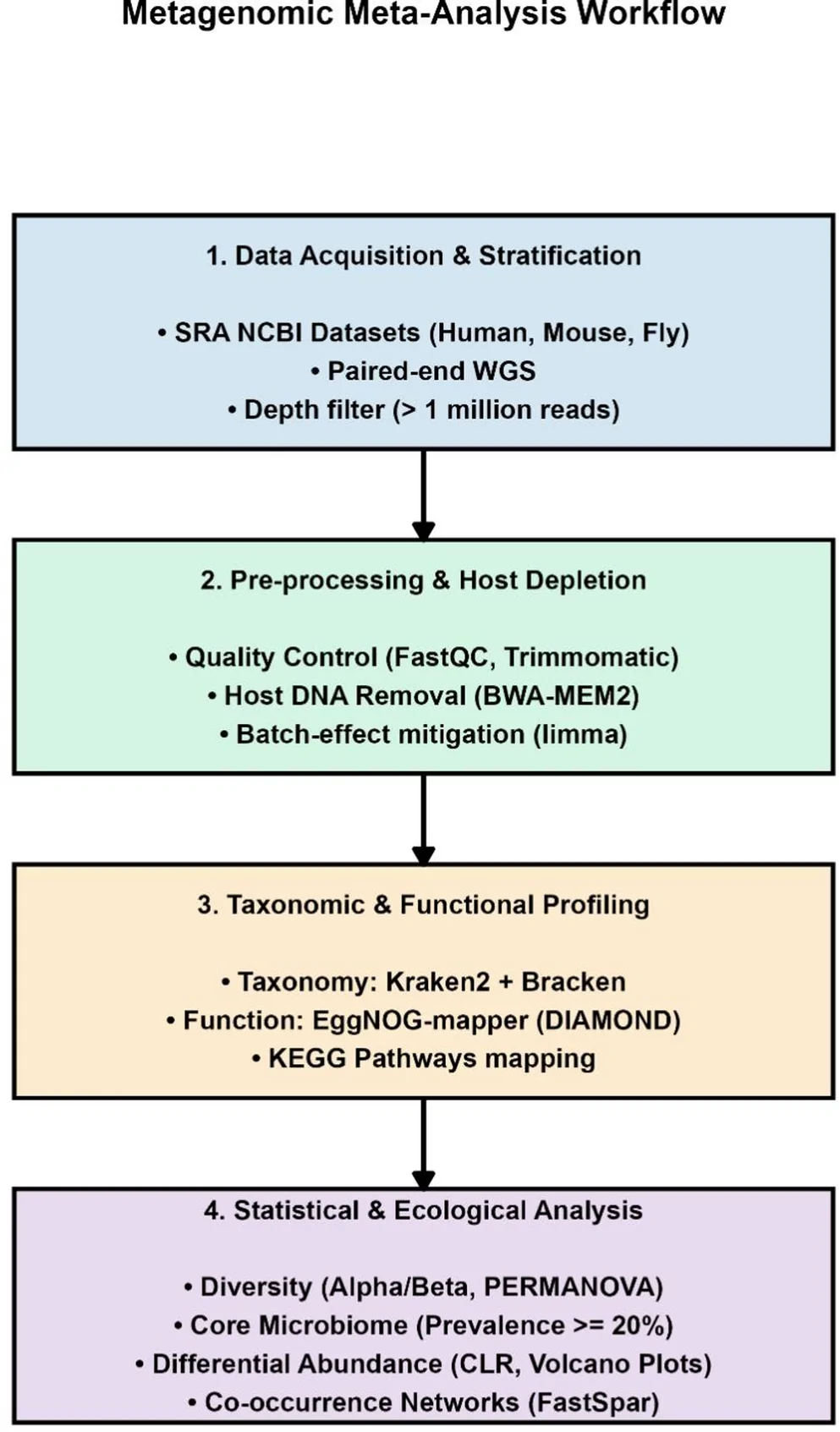
Schematic overview of the analytical workflow. The diagram illustrates the four primary phases of the cross-species meta-analysis: (1) Data acquisition and strict inclusion criteria for *Homo sapiens*, *Mus musculus*, and *Drosophila melanogaster* datasets; (2) Bioinformatic pre-processing, including host DNA depletion and batch-effect mitigation; (3) Taxonomic and functional metagenomic profiling; and (4) Downstream statistical and ecological analyses, encompassing diversity metrics, core microbiome characterisation, and differential abundance of conserved metabolic signatures

A schematic overview of the analytical workflow is presented in Fig. 1. Raw sequencing reads underwent a standardised quality control process—including adapter trimming using Trim Galore (v0.6.10)—and host DNA removal through alignment against the reference genomes of *Homo sapiens* (GRCh38.p14), *Mus musculus* (GRCm39), and *Drosophila melanogaster* (BDGP6) using BWA-MEM2 (v2.2.1). A detailed description of these pre-processing protocols, alongside the specific criteria for sample exclusion and the handling of sequencing depth artefacts, is provided in the Supplementary Methods (Sect.  1.1 to 1.4).

To minimise potential technical artefacts arising from heterogeneous datasets, batch-effect (Sect.  1.5) correction was performed using the *removeBatchEffect* function implemented in the limma (Ritchie et al. 2015) R package, while preserving biological variance through the inclusion of an appropriate design matrix. Visual evaluation of the batch-effect correction is available in Supplementary Figure S1.

### Taxonomic Classification and Diversity Analysis

Taxonomic classification at the species and genus levels was performed using Kraken2 (v2.1.3) (Wood et al. 2019) and Bracken (Lu et al. 2022) against the Kraken2 Standard database. To estimate the ecological complexity of the microbial communities (α-diversity), the Chao1 (Chao and Lee 1992), Shannon (Shannon 1948), and Simpson (Simpson 1949) indices were calculated using unnormalised count tables to preserve the abundance structure of rare taxa. Given non-normal data distributions (assessed via Shapiro-Wilk tests), global and pairwise differences in α-diversity across host species and sexes were evaluated using Kruskal-Wallis and Wilcoxon rank-sum tests, respectively, with Bonferroni correction for multiple comparisons.

To address the compositional nature of microbiome sequencing data, standard distance metrics on raw counts were avoided (Gloor et al. 2017). Instead, species abundance matrices underwent Centred Log-Ratio (CLR) transformation to enable standard multivariate statistics. β diversity (sample dissimilarity) was visualised using Principal Component Analysis (PCA) based on Aitchison distance (Aitchison 1982; Aitchison et al. 2000). The statistical effects of host species identity and biological sex on community structure were evaluated via Permutational Multivariate Analysis of Variance (PERMANOVA) with 9,999 permutations using the adonis2 function. All ecological and statistical analyses were conducted in R utilising the phyloseq, vegan, and tidyverse packages, with significance defined as *p* < 0.05.

### Microbial Co-occurrence Networks and Keystone Taxa

Ecological interactions were initially inferred using FastSpar (Watts et al. 2019), an optimized implementation of SparCC (Friedman and Alm 2012), with 1,000 bootstrap permutations. For general topological characterization, interactions were retained using a correlation threshold of |r| > 0.5 to mitigate potential artifacts without over-penalizing biological associations in high-diversity microbiomes. Topological metrics were characterized using the igraph package (Ning et al. 2022).

To identify and delineate the global core interactome (keystone taxa) across all hosts, a highly stringent unified network was constructed. Count data was center-log ratio (CLR) transformed to account for compositionality, and a prevalence filter of ≥ 10% was applied alongside the removal of known environmental contaminants (defined as taxa predominantly identified within the negative sequencing controls and well-documented DNA extraction kit artifacts) to ensure biological validity. Robust structural interactions were retained applying a Spearman correlation threshold of |r| > 0.70. To ensure continuous topological cohesiveness, the giant component of the network was extracted. Community structure and modularity were resolved via the Walktrap clustering algorithm. Finally, the top 200 nodes exhibiting the highest degree centrality were visualized using a manually compressed force-directed layout (Fruchterman-Reingold) in ggraph (Pedersen 2025) to map the physical integration of the primary functional modules.

### Core Microbiome and Universal Differential Abundance

To delineate shared and host-specific taxa across *H. sapiens*, *M. musculus*, and *D. melanogaster*, intersection analyses were performed. A prevalence filter was applied to mitigate transient noise (Shade and Handelsman 2012), retaining only species with non-zero abundance across the aggregated datasets. Taxonomic intersections were calculated using ggVennDiagram (Gao et al. 2021). To assess host-specific ecological preferences within the Universal Core, batch-corrected CLR abundances for the 15 intersecting species were averaged per host and visualised via hierarchical clustering using the pheatmap package (Kolde 2019).

### Functional Profiling and Differential Metagenomic Analysis

Metagenomic functional potential was characterized using EggNOG-mapper (v2) (Cantalapiedra et al. 2021). Paired-end reads were aligned against the EggNOG 5.0 database using DIAMOND (v2.1.8) (Buchfink et al. 2021; Huerta-Cepas et al. 2019). Gene families were mapped to KEGG pathways utilising the KEGGREST (v1.42.0) package) (Kanehisa et al. 2017; Tenenbaum and Maintainer 2021), and aggregated pathway abundances were CLR-transformed using the compositions (v2.0-6) package (Susin et al. 2020). Global metabolic architecture was visualised via unsupervised hierarchical clustering and PCA. Differential pathway abundance between hosts was evaluated using empirical Bayes moderation in limma (Ritchie et al. 2015). For the Mouse vs. Fly contrast, one low-depth murine sample (SRR10905282) was excluded to prevent zero-inflation artefacts. Significant pathways (nominal p-value < 0.05, |log2 FC| > 1) were visualized via Volcano Plots. To elucidate taxon-function associations, Spearman’s rank correlation linked differentially abundant pathways to specific taxa, defining primary drivers as species with ρ > 0.6. In this study, the conserved mammalian metabolic core is operationally defined and quantitatively assessed as the intersecting set of fundamental KEGG pathways (e.g., central carbon metabolism and essential amino acid biosynthesis) that maintain high cumulative relative abundance and exhibit no statistically significant differential abundance (padj > 0.05) between human and murine hosts.

### Cross-Species Trend Analysis of Sex-Dimorphism

To investigate evolutionarily conserved sex-specific signatures across mammalian hosts, an algebraic trend alignment approach was employed due to murine sample size constraints (which precluded conventional intra-species variance estimation). The degree of sexual dimorphism was quantified as the log_2_ Fold Change (CLR _Male_ - CLR _Female_) for each KEGG pathway. A metabolic pathway was classified as a ‘Putative Conserved Sex Signature’ only if it exhibited both directional concordance (identical enrichment sign in humans and mice) and sufficient biological magnitude across both species. This vector-based alignment filters stochastic host-specific noise, isolating parallel evolutionary metabolic trajectories.

### Genomic Divergence Analysis

To assess the taxonomic independence of the microbial communities identified across the three hosts, a comparative genomic distance analysis was performed. A total of 116 representative reference genomes (NCBI RefSeq) were analysed. These reference genomes were selectively chosen to encompass the dominant taxonomic diversity previously identified across the three-host species within our dataset, providing a robust genomic baseline for comparative distance estimation. Global genomic distances were estimated using the MinHash algorithm implemented in Mash v2.3 (Ondov et al. 2016) employing a sketch size of 1,000 hashes and a k-mer length of 21. The resulting Jaccard distance matrix was processed in R v4.2 using the ComplexHeatmap package (Gu 2022; Gu et al. 2016) Hierarchical clustering was executed via the Ward.D2 method to identify patterns of genomic similarity. Furthermore, the 95% Average Nucleotide Identity (ANI) threshold was adopted as the biological boundary for species delimitation, as established by recent large-scale genomic surveys (Jain et al. 2018).

## Results

### Validation of Sampling Robustness

Rarefaction analysis confirmed that taxonomic discovery reached a stable plateau across all host species (Supplementary Figure S2). For the murine dataset, the accumulation curve reached an asymptote well before the total sample size (n = 75), with a final slope of 0.1498. This indicates that the probability of detecting new taxa with additional sampling is minimal, ensuring that the ‘core interactome’ is robustly represented (Chao and Jost 2012). These results validate the integrity of the subsequent comparative analyses, confirming that observed structural divergences are biological in origin rather than artefacts of sampling depth.

### Inter-host Microbial Communities Exhibit High Genomic Divergence

Mash distance analysis revealed a pronounced genomic divergence among the microorganisms associated with the three hosts. The vast majority of pairwise comparisons between different hosts yielded a Mash distance (*D*) significantly exceeding the established species-boundary threshold (*D* > 0.05), which corresponds to an ANI of < 95%(Jain et al. 2018). These findings demonstrate that, despite sharing 123 genera (representing 7.55% of the total 1,629 genera identified across the mammalian cohort at a 20% prevalence threshold), the specific microbial strains comprising the co-occurrence networks of each host are genomically distinct. Consequently, the reported microbiome architectures are host-specific and not confounded by taxonomic redundancy (Figure S3).

### Ecological Filtering Drives Taxonomic and Structural Divergence Across Hosts

**Fig. 2:**
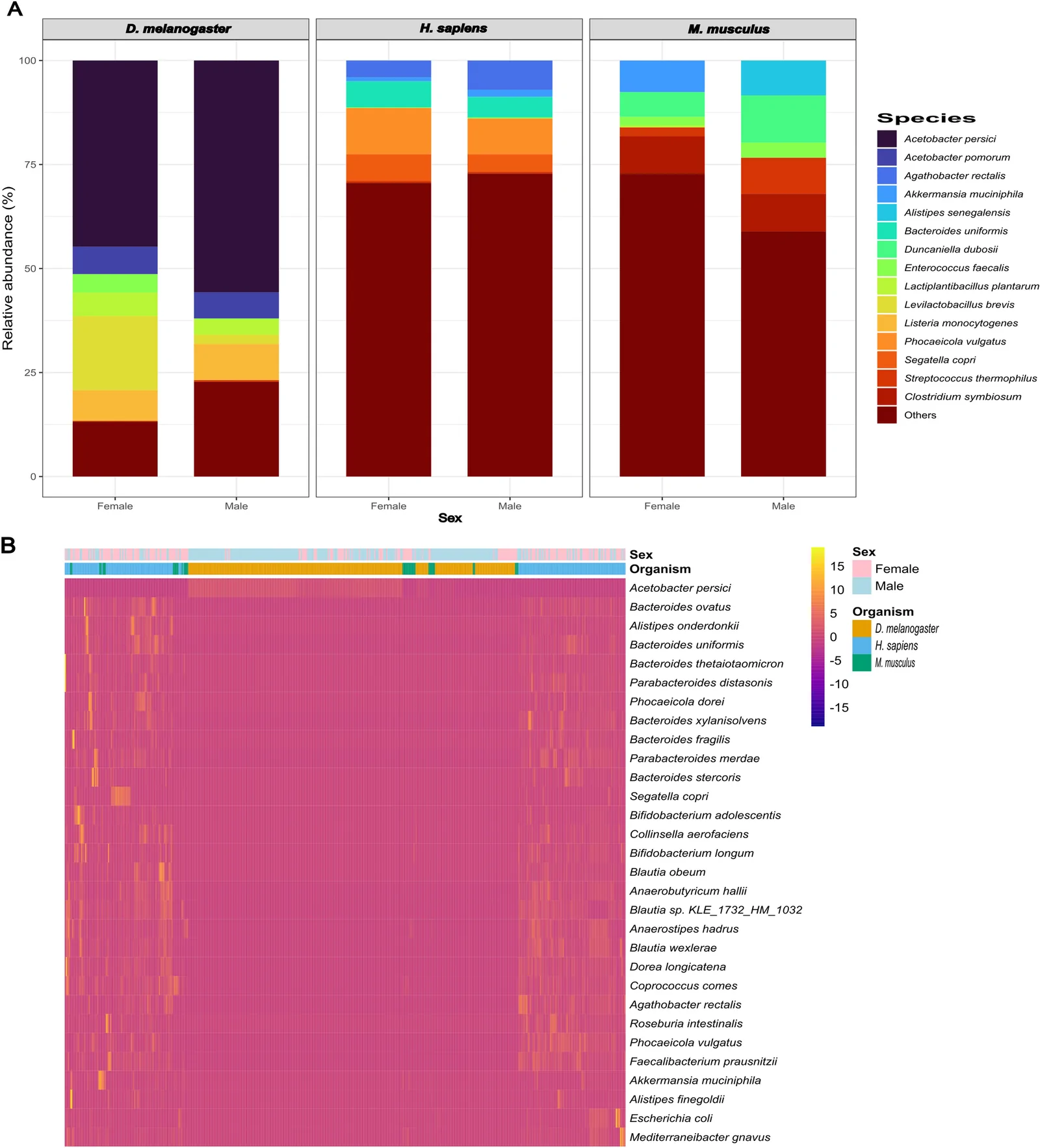
**A** Taxonomic profiling and relative abundance of dominant bacterial species across host organisms and sex. Stacked bar plots represent the mean relative abundance (%) of the top 15 bacterial species—defined by the highest mean relative abundance across all samples—stratified by host and sex. All remaining lower-abundance taxa are aggregated into the “Others” category to prevent colour palette exhaustion. Crucially, all formal alpha diversity statistics were calculated on the complete, un-agglomerated feature matrices prior to this visual grouping. The microbiota of *D. melanogaster* is dominated by *Acetobacter* and *Lactobacillus* species, whereas human and mouse profiles exhibit greater diversity with substantial contributions from the “Others” category. **B** Heatmap of core bacterial community profiles. The heatmap visualises the Centred Log-Ratio (CLR) abundance (Aitchison distance) of the 30 targeted core bacterial species across all samples. Columns correspond to individual samples grouped by host species, while rows represent bacterial taxa. Top annotations indicate host identity using a standardised colour coding (*Homo sapiens* in blue, *Mus musculus* in green, and *Drosophila melanogaster* in orange) and biological sex (females in pink, males in light blue). Colour intensity for bacterial abundance follows the “plasma” scale, ranging from dark purple (low abundance/absent) to bright yellow (high abundance). Distinct clusters highlight host-specific bacterial signatures and the mutual exclusion between insect and mammalian microbiomes, defining three distinct community profiles driven by host identity (Color figure online)

Analysis of taxonomic composition (Fig. 2A) where we visualised the top 15 bacterial species (defined operationally as those with the highest mean relative abundance across the entire dataset), revealed significant differences in dominant bacterial species among the three model organisms. The *Drosophila melanogaster* microbiome exhibited a low-complexity structure, dominated almost exclusively by members of the genera *Acetobacter* and *Lactobacillus*, which constituted most of the relative abundance, leaving a minor fraction (< 5%) for the “Other” category. This low diversity contrasts with mammals, where the “Other” category represented a considerable proportion of the community, reflecting a higher cumulative relative abundance of the low-abundance microbial community.

Species within the genus *Bacteroides* showed high prevalence in both mammalian hosts, being detected in 98.2% of human samples and 84.5% of murine samples, a finding consistent with its identification as a core member of the mammalian gut microbiota (Falony et al. 2016; Nguyen et al. 2015). Conversely, the genus remained virtually absent (< 1%) in *D. melanogaster*, aligning with reports that the dipteran gut is dominated by *Acetobacter* and *Lactobacillus*, typically lacking the Bacteroidetes phylum (Douglas 2018; Chandler et al. 2011). Although 4 species were shared between *H. sapiens* and *M. musculus* (including *B. uniformis* and *B. vulgatus*), they exhibited contrasting abundance patterns; for instance, *B. uniformis* reached a mean relative abundance of 12.4% in humans compared to only 1.2% in mice. This disparity highlights host-specific ecological niches and suggests that while taxonomic overlap exists at the genus level, the relative contribution of species is strongly governed by the host’s physiological environment (Hugenholtz and de Vos 2017; Ley et al. 2005).

Heatmap visualization (Fig. 2B) confirmed the existence of three distinct community profiles. The robust segregation of these profiles was not merely visual, but mathematically driven by unsupervised hierarchical clustering (Ward.D2) on Aitchison distances (Murtagh and Legendre 2014) and statistically validated by our global PERMANOVA model. A cluster of bacteria highly specialized to *Drosophila* (bright yellow zone in fly columns) was identified, which was completely absent (dark purple zone) in mammals. Similarly, a core of bacteria shared between humans and mice showed mutual exclusion patterns with the insect microbiome, corroborating that broad host phylogeny strongly correlates with bacterial community structure. Nevertheless, it is essential to acknowledge that in macro-ecological meta-analyses, ‘host identity’ functions as a composite variable; it encapsulates not only evolutionary genetics but also inextricably linked biological factors such as species-specific diets, gastrointestinal physiologies, and environmental exposures (e.g., controlled laboratory versus free-living conditions). While cross-study technical variance was rigorously mitigated via our limma-based batch correction, these intrinsic biological and dietary confounders collectively define the host-specific ecological niche. No drastic changes in dominant species composition associated with sex were detected, suggesting that the richness differences previously observed in female vs. male flies may be driven by low-abundance satellite species rather than a turnover of core symbionts.

**Fig. 3:**
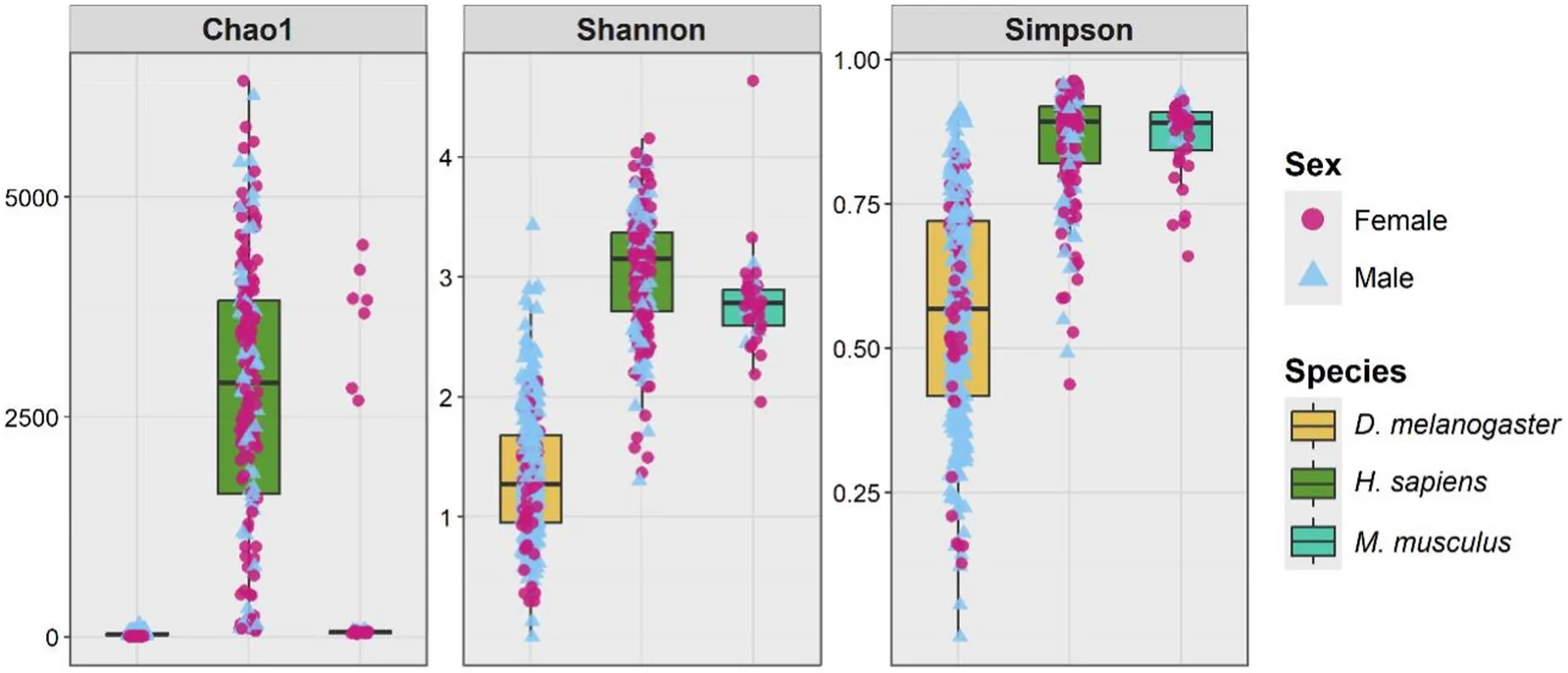
Impact of host phylogeny and sex on microbial community complexity (Alpha Diversity). Within-sample diversity was estimated using Chao1, Shannon, and Simpson indices. Mammals (*H. sapiens* and *M. musculus*) exhibited significantly higher diversity and community complexity compared to the model insect *D. melanogaster* (Kruskal-Wallis, *p* < 0.001). Although humans and mice differ in species richness, they share a similar dominance structure (Simpson Index, *p* = 0.85). Sex-stratified analysis (Wilcoxon test) revealed that community structure is stable between males and females in mammals; however, significant sexual dimorphism was observed in *Drosophila*, where sex influences the richness of harboured bacterial species (*p* < 0.001)

α diversity analysis revealed that host species identity is the primary determinant of gut microbiome complexity (Kruskal-Wallis, *x*^2^ > 340, *p* < 2.2 × 10^− 16^ for all evaluated indices). A diversity gradient was observed wherein mammals (*Homo sapiens* and *Mus musculus*) harbour significantly richer and more diverse communities compared to the model insect *Drosophila melanogaster*. Regarding Observed Richness and Shannon Index, significant differences were found between all species pairs (Wilcoxon post-hoc, *p* < 0.001). Humans exhibited the highest richness and evenness (Chao1: 4,452.1 ± 1,215.3; Shannon: 3.52 ± 0.42), followed by mice (Chao1: 184.5 ± 45.2; Shannon: 3.11 ± 0.35), while *Drosophila* presented markedly reduced diversity (Chao1: 38.2 ± 12.1; Shannon: 1.18 ± 0.28). These values represent the average metrics across all high-quality samples included for each species (*H. sapiens*, *n* = 202; *M. musculus*, *n* = 75; *D. melanogaster*, *n* = 243). However, analysis of the Simpson Index revealed an interesting pattern of ecological convergence: although humans and mice differ in richness, no significant differences were found in their dominance structure (*p* = 0.85) (Fig. 3). This indicates that, despite taxonomic divergence, no statistically significant difference in functional dominance was detected between the mammalian hosts, which differs drastically from the high dominance observed in *Drosophila* (*p*_*adj*_ < 2 × 10^− 16^).

**Fig. 4:**
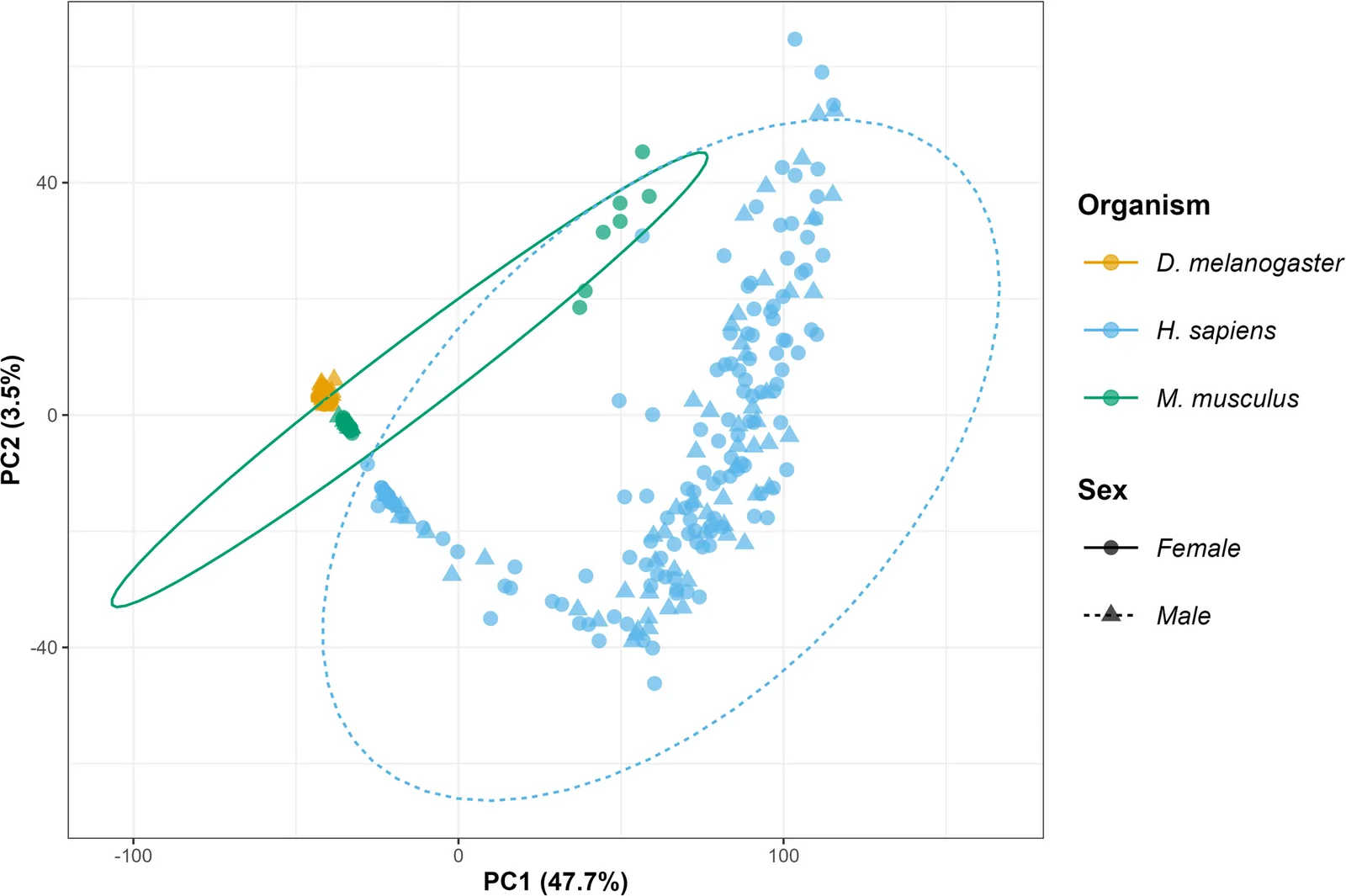
Principal Component Analysis (PCA) of microbial community clustering by host organism and sex. Data points represent individual gut microbiome samples coloured by host species: *Homo sapiens* (blue), *Mus musculus* (green), and *Drosophila melanogaster* (orange). Point shapes denote biological sex: circles indicate females and triangles indicate males. The proportion of total variance explained by each principal component is indicated on the axes (PC1: 47.2%; PC2: 3.5%). Ellipses represent 95% confidence intervals around the group centroids (*N* = 520) (Color figure online)

To evaluate the variation in gut microbiota composition among different host species (*Homo sapiens*, *Mus musculus*,* Drosophila melanogaster*) and sexes, a Principal Component Analysis (PCA) was performed on Centered Log-Ratio (CLR) transformed data. The resulting ordination (Fig. 4) displays a clear separation of samples driven primarily by host identity. The first two principal components (PC1 and PC2) jointly explain 50.7% of the total data variance. A distinct clustering pattern is observed where *D. melanogaster* samples segregate strongly from mammals (*Homo sapiens* and *Mus musculus*) along the first component (PC1), reflecting substantial phylogenetic and physiological differences in their microbiomes. Meanwhile, humans and mice, although clustering closer together, form discrete groups primarily separable along the second component (PC2). The statistical significance of these observations was confirmed via Permutational Multivariate Analysis of Variance (PERMANOVA). The global model revealed highly significant differences in microbial community structure attributable to species and sex variables (*Pseudo*-F_3,514_= 105.83, *p* < 0.001). Notably, these variables explain a substantial portion of the observed biological variability (R^2^ = 0.3818), indicating that over 38% of the variation in taxonomic composition is determined by the biological identity and sex of the host.

### Global Differential Patterns

Differential abundance analysis of KEGG pathways confirmed that host phylogeny is the primary determinant of the microbiome’s functional repertoire. To dissect these functional shifts, three evolutionary contrasts were evaluated.

### The Mammalian Functional Core (Human vs. Mouse)

**Fig. 5:**
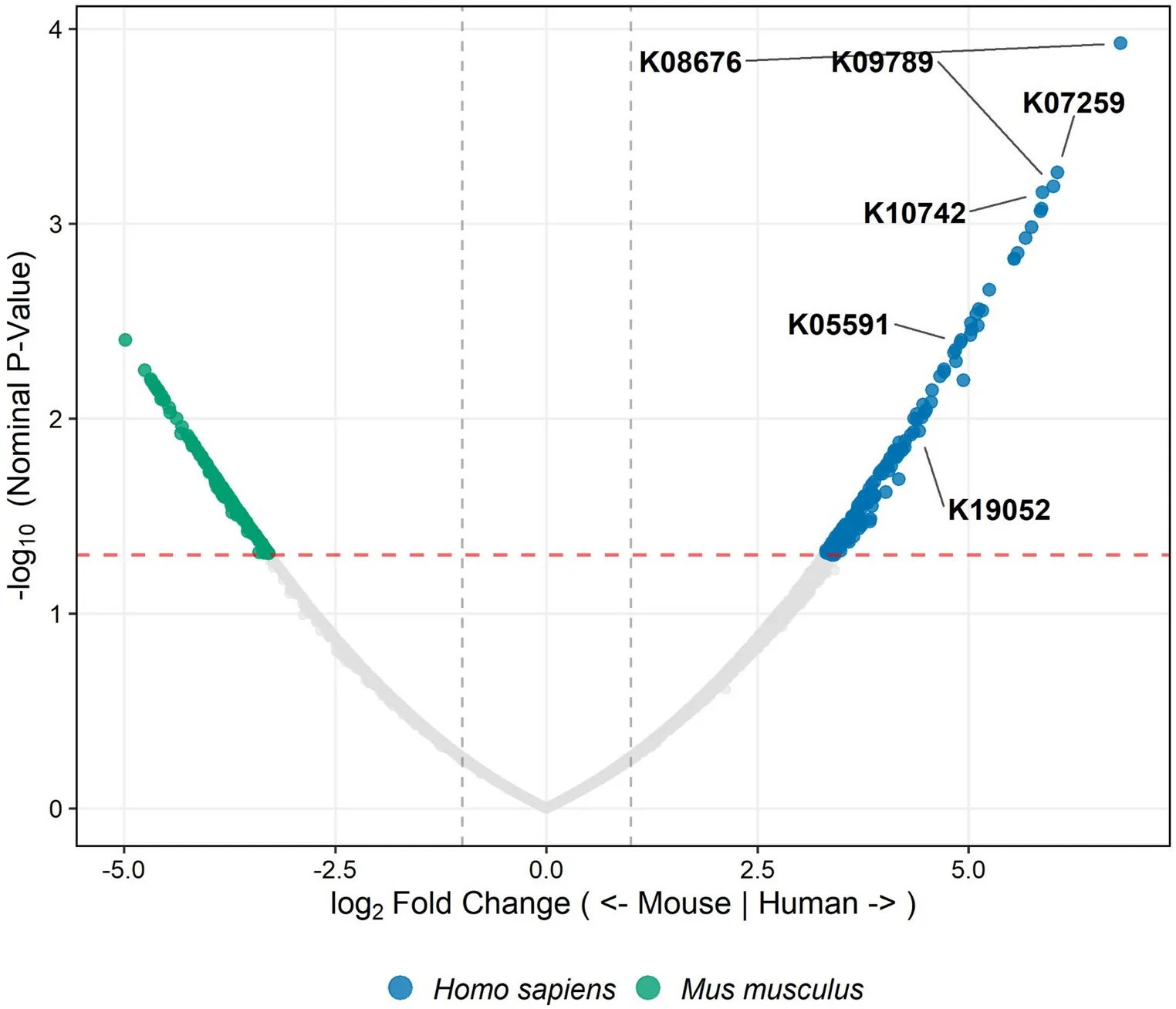
Functional divergence between mammalian hosts. Volcano plot of KEGG Orthology (KO) profiles between *Homo sapiens* (blue data points) and *Mus musculus* (green data points) reveals a substantial shared functional core, accounting for 69.37% of the total detected KOs. Statistical validation using Aitchison distances confirms that inter-host functional divergence is comparable to intra-host variation (Bastiaanssen et al. 2023). The strong correlation in KO relative abundances (Spearman’s ρ = 0.74, *p* < 0.001) indicates a topographically conserved metabolic architecture across mammalian models, primarily driven by central metabolism and essential biosynthesis pathways. Despite this baseline conservation, species-specific functional adaptations are evident. For visual clarity, only a subset of the most statistically significant divergent KEGG modules (defined by an absolute log_2_Fold Change > 1 and a nominal p-value < 0.05) are explicitly annotated. These selective labels highlight the most pronounced metabolic specialisations enriched in either the human or murine microbiome (Color figure online)

The comparison between the mammalian hosts (Fig. 5) delineates a shared functional core centred on fundamental metabolic requirements. Analysis of the KEGG Orthology (KO) profiles identified that 69.37% of the total KOs are shared between the human and mouse datasets, constituting a robust functional baseline (Turnbaugh et al. 2007). Quantification of the functional landscape using Aitchison distances further corroborated this congruence; inter-host functional dissimilarity (e.g., Human Male vs. Mouse Female, *D* = 148.6) was found to be remarkably similar to intra-host variation within the human cohort (*D* = 141.3), suggesting a highly conserved metabolic backbone (Bastiaanssen et al. 2023). Despite taxonomic differences, the overall functional architecture remains comparable in terms of metabolic scaling; a correlation analysis of KO abundances yielded a Spearman’s ρ of 0.74 (*p* < 0.001), indicating that fundamental biological roles are topographically conserved across these mammalian models.

However, superimposed on this conserved core are species-specific metabolic signatures. To highlight these functional disparities, a subset of the most statistically significant and biologically relevant KEGG modules were annotated in Fig. 5. In *Homo sapiens*, the functional profile was characterised by a significant enrichment in modules such as DNA replication and repair (e.g., K10742) and biotin metabolism (e.g., K09789). Conversely, the metabolic landscape in *Mus musculus* exhibited distinct adaptive signatures, fully described in Supplementary Table S3. The selective labelling of these specific modules illustrates the domain-specific functional divergence that occurs despite the overarching macro-metabolic conservation.

### The Vertebrate-Invertebrate Split (Human vs. Fly)

**Fig. 6:**
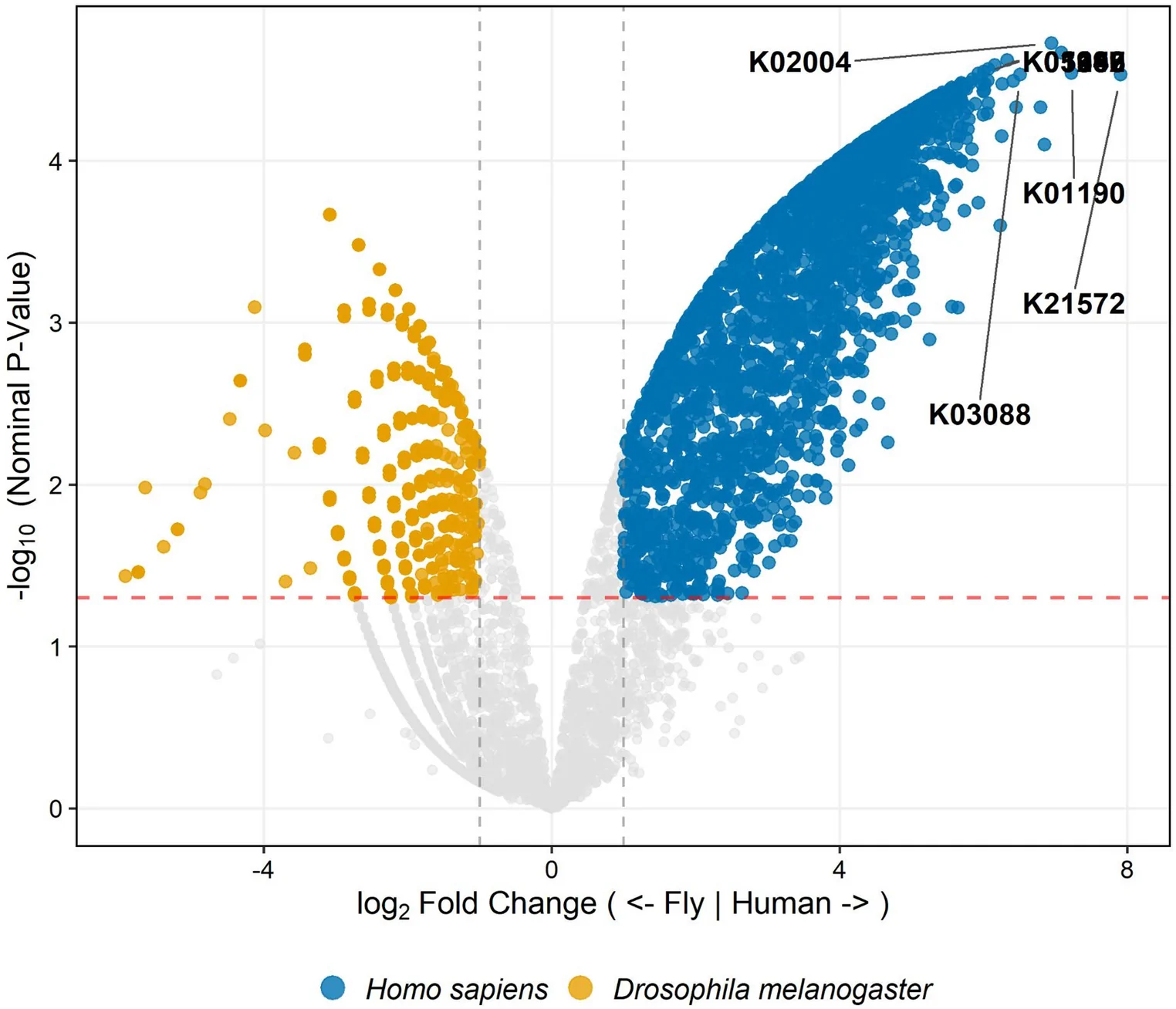
Evolutionary functional gap between Human and Fly. This differential functional profiling (KEGG Orthology) between *Homo sapiens* and *Drosophila melanogaster* reveals a pronounced asymmetry in metabolic potential, underpinned by a near-total functional discontinuity characterised by a shared core of only 0.62% and a negligible Spearman’s correlation (ρ = 0.01, *p* < 0.001). The plot highlights distinct enrichment patterns where blue points (positive log2 FC) denote the extensive repertoire of multifaceted metabolic and signalling functions enriched in the human microbiome, while orange points (negative log2 FC) represent the specialised and limited set of functions enriched in the fly, primarily associated with primary metabolic maintenance. These results, calculated using Aitchison distances within the compositional framework of Bastiaanssen et al. (2023), illustrate a functional split that reflects the deep phylogenetic and physiological distance between vertebrate and invertebrate hosts.(Brooks et al. 2016; Ley et al. 2008). For visual clarity, only a highly significant subset of human-enriched KEGG modules (absolute log2 FC > 1; nominal p-value < 0.05) are explicitly annotated on the plot, whilst the complete list of modules significantly enriched in the *Drosophila* microbiome is provided in Supplementary Table S3 (Color figure online)

In contrast to the mammalian models, the comparison between *H. sapiens* and *D. melanogaster* reveals a pronounced functional discontinuity. Quantitatively, the shared functional core is virtually non-existent, comprising only 0.62% of the total KOs. This divergence is further substantiated by a negligible Spearman’s correlation (ρ = 0.01, *p* < 0.001), indicating a lack of metabolic scaling (Fig. 6).

Differential enrichment analysis shows that human-associated communities are uniquely equipped for host-microbiome signalling and endocrine support. Specifically, we identified an extensive repertoire of KOs for steroid hormone biosynthesis (e.g. K00067), secondary bile acid metabolism (e.g. K01442), and tryptophan metabolism (e.g. K01652, K01653), which are essential for vertebrate systemic homeostasis. Conversely, the *D. melanogaster* microbiome is functionally streamlined; its metabolic potential is primarily restricted to essential maintenance, with dominant KOs being limited to basic cellular machinery such as ribosomal proteins (e.g. K02950, K02946). This split reflects the transition from the transient, simple microbiota of invertebrates to the complex, homeostatic functional core of mammals (Brooks et al. 2016; Douglas 2018).

### Divergence of Model Organisms (Mouse vs. Fly)

**Fig. 7:**
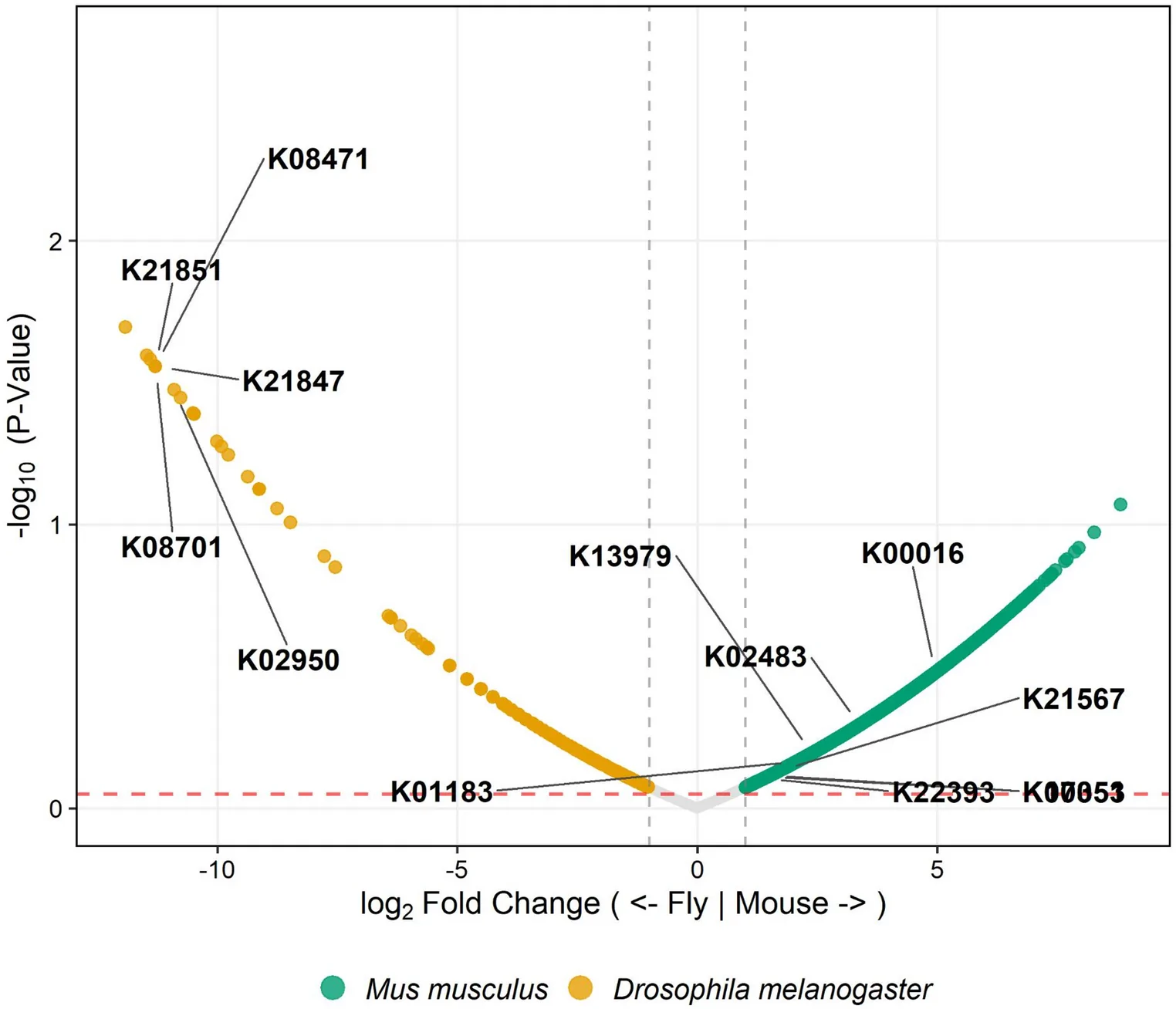
Functional divergence of model organisms (Mouse vs. Fly). Comparison of the functional landscape between *Mus musculus* and *Drosophila melanogaster*. Green points (positive log_2_ FC) indicate pathways significantly enriched in the murine microbiome, including vertebrate-specific endocrine and immune support functions. Orange points (negative log2 FC) denote pathways enriched in the fly. The explicit separation (defined by |log2 FC| > 1 and nominal p-value < 0.05) underscores the limited translational applicability of the invertebrate microbiome for modelling mammalian systemic traits. Labeled KOs represent specific enriched metabolic pathways discussed in the text, alongside top divergent functional markers detailed in Supplementary Table S3 (Color figure online)

Finally, the direct comparison between the two laboratory models (Fig. 7) confirmed that the murine microbiome possesses a significantly higher functional density. The comparison between the two most widely used model organisms, *M. musculus* and *D. melanogaster*, highlights a significant functional divergence that limits their interchangeability in microbiome research. Quantitatively, the shared functional core between these models is negligible, representing only 0.78% of the total KOs identified. This lack of congruence is further demonstrated by a near-zero Spearman correlation (ρ = 0.018, *p* < 0.001), indicating that their microbial communities operate under independent metabolic frameworks. The mean Aitchison distance (*D* = 272.41) between mouse and fly profiles significantly exceeds the variation observed between mammals, statistically validating the “model organism gap”.

Differential analysis revealed that the fly microbiome is uniquely enriched in pathways tailored to the insect physiological environment. Specifically, we identified enrichment in chitin degradation (e.g. K01183) and acetic acid metabolism (e.g. K00016), which are essential for *Drosophila* energy homeostasis but sparsely represented in the murine gut. Furthermore, signalling pathways such as the two-component system (e.g. K02483), involved in the regulation of insect-specific gut colonisation, were characteristically enriched in fly datasets. In contrast, the mouse microbiome exhibits a functional density dedicated to complex carbohydrate fermentation and vertebrate-specific bile acid modulations(Ley et al. 2008), confirming that these models represent distinct evolutionary solutions to host-microbe symbiosis.

### Effect of Sex in Microbiome Communities

Upon evaluating the influence of sex on alpha diversity, host-dependent differential behaviour was observed. In mammalian models (*H. sapiens* and *M. musculus*), no significant differences were detected between males and females in any diversity index (*p* > 0.05), indicating a sex-independent structural stability of the microbiome. In contrast, *Drosophila melanogaster* presented significant sexual dimorphism restricted to Observed Richness (*p* = 5.91 × 10^− 12^), where a consistent difference in the number of species carried by males and females was detected. Nevertheless, this difference in richness did not translate into significant changes in evenness (Shannon, *p* = 0.09) or dominance (Simpson, *p* = 0.07), suggesting that the sex effect in flies primarily impacts the presence of low-abundance or transient taxa without altering the core structure of the dominant community. This claim is statistically supported by the lack of significant sexual dimorphism in Shannon evenness (*p* = 0.09) and Simpson dominance (*p* = 0.07), despite the marked differences observed in total richness (*p* < 0.001). Quantitatively, the dominant core, composed of the genera *Acetobacter* and *Lactobacillus*, maintained a stable high prevalence, collectively accounting for over 95% of the total relative abundance in both sexes, with less than a 1% difference in their combined mean abundance between males and females.

### Host-Specific Network Structure

**Fig. 8:**
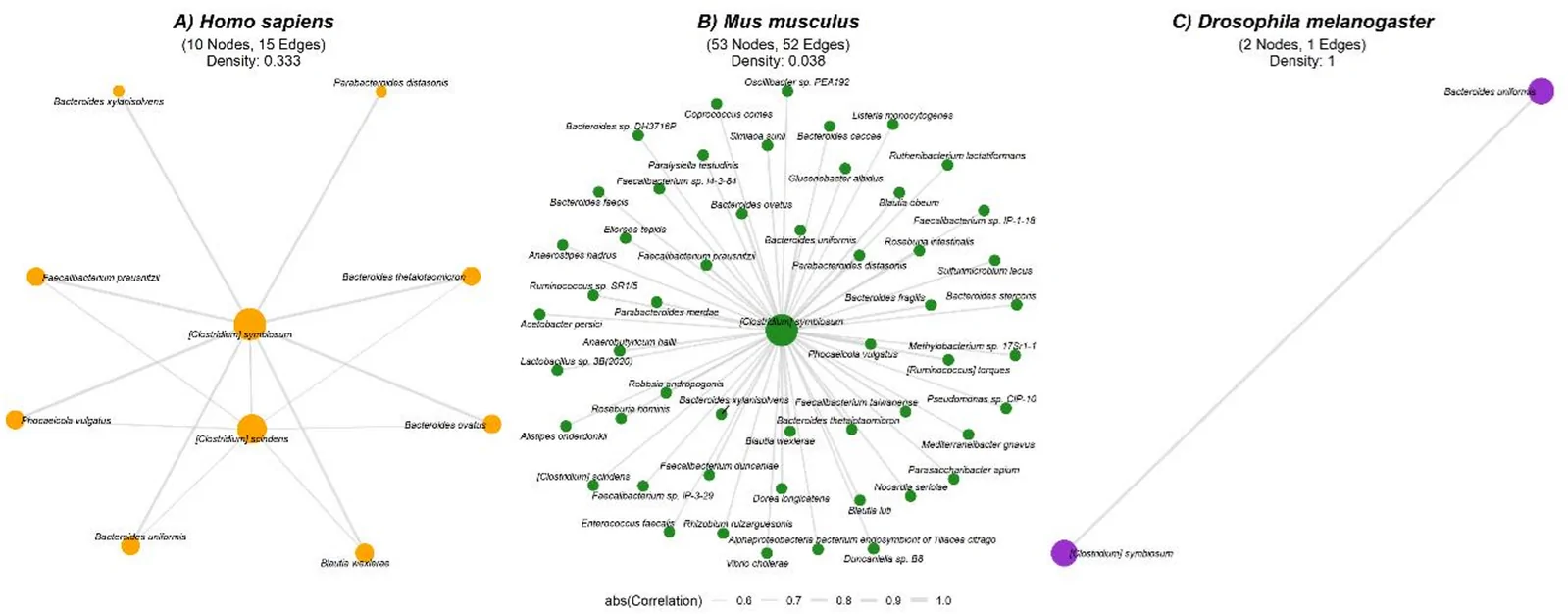
Comparative topology of microbial co-occurrence networks across host species. Network visualizations based on FastSpar correlation inference for **A** Human, **B** Murine (Mouse), and **C** Fruit Fly microbiomes. Nodes (circles) represent bacterial species, and edges (grey lines) indicate strong ecological interactions (|r| > 0.5). Node size is proportional to degree centrality (number of direct connections). Topological metrics (number of Nodes, Edges, and Network Density) are provided for each host-specific network (Color figure online)

Topological analysis revealed distinct structural complexities across the three-host species (Fig. 8). The *Mus musculus* microbiome exhibited the most robust and structured network (53 nodes, 61 edges; modularity = 0.283), suggesting a defined community organization. Conversely, the *Homo sapiens* network was smaller (10 nodes, 15 edges) yet exhibited significantly higher density (0.333 vs. 0.044 in mice), indicating that while fewer species maintain consistent correlations across individuals, those that do form a highly cohesive core structure. The *Drosophila melanogaster* network was minimal (2 nodes, 1 edge), reflecting a simple ecosystem dominated by binary interactions (Fig. 8). From an evolutionary network perspective, these topological disparities reflect distinct adaptive strategies. The highly modular structure of the *Mus musculus* interactome is characteristic of evolutionarily robust biological systems, where functional compartments prevent localised ecological perturbations from causing systemic collapse (Barabási and Oltvai 2004). In contrast, the high-density human network suggests a densely co-evolved guild of keystone taxa heavily reliant on cross-feeding metabolic dependencies, which maximises metabolic efficiency but may increase susceptibility to cascading extinctions (Faust et al. 2012).

### Global Interactome and Keystone Taxa

**Fig. 9:**
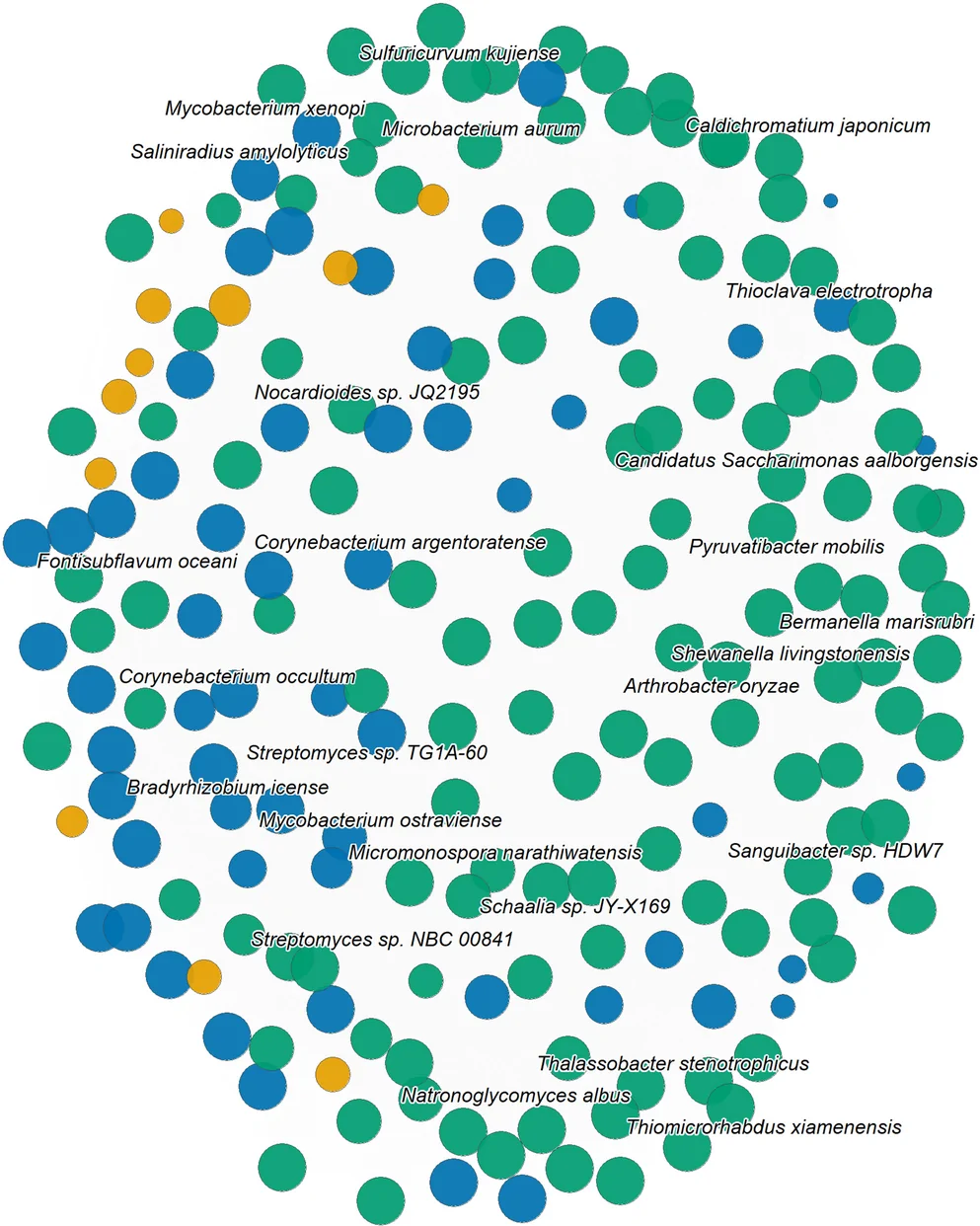
Global co-occurrence network and core interactome analysis. The network integrates samples across host species, focusing on the giant component of the ecosystem and highlighting the top 200 structural hubs based on degree centrality. Robust ecological interactions (defined by a stringent Spearman |r| > 0.70 threshold following CLR transformation) determine the spatial topology of the network. Due to the high interconnectivity of the core interactome, explicit edge lines have been omitted to maintain visual clarity; instead, interaction strength is reflected by spatial proximity. Node size is proportional to the degree of connectivity, while distinct node colours (e.g., purple, green, orange) denote separate modular sub-communities identified via the Walktrap clustering algorithm. Labels indicate the most highly connected keystone taxa. The network core is dominated by a synergistic consortium of primary complex carbohydrate degraders (e.g., *Bacteroides spp*., *Phocaeicola vulgatus*) and prominent butyrate-producing bacteria (e.g., *Faecalibacterium*, *Roseburia*, *Agathobacter*), emphasizing their foundational role as ecological drivers across mammalian gut microbiomes (Color figure online)

To delineate the ecosystem backbone, the giant component of the global network was extracted, and the top 200 structural hubs (Fig. 9) were analysed using a robust correlation threshold (|r| > 0.70). The refined network core was dominated by a synergistic functional guild composed of complex carbohydrate degraders and Short-Chain Fatty Acid (SCFA) producers, reflecting highly conserved metabolic cross-feeding networks. Specifically, members of the Bacteroidaceae family, including *Bacteroides uniformis*, *Bacteroides ovatus*, *Bacteroides thetaiotaomicron*, and *Phocaeicola vulgatus*, emerged as primary keystone taxa alongside major butyrate-producing bacteria. Species exhibiting the highest degree of centrality also included prominent Firmicutes such as *Faecalibacterium duncaniae*, *Faecalibacterium prausnitzii*, *Agathobacter rectalis*, and *Roseburia intestinalis*, as well as *Dysosmobacter welbionis*. The high centrality of these taxa indicates that primary glycan degradation coupled with downstream butyrate production acts as the foundational ecological driver, orchestrating community assembly and maintaining homeostasis across mammalian gut microbiomes.

### Taxonomic Intersection and the “Mammalian Signature”

**Fig. 10:**
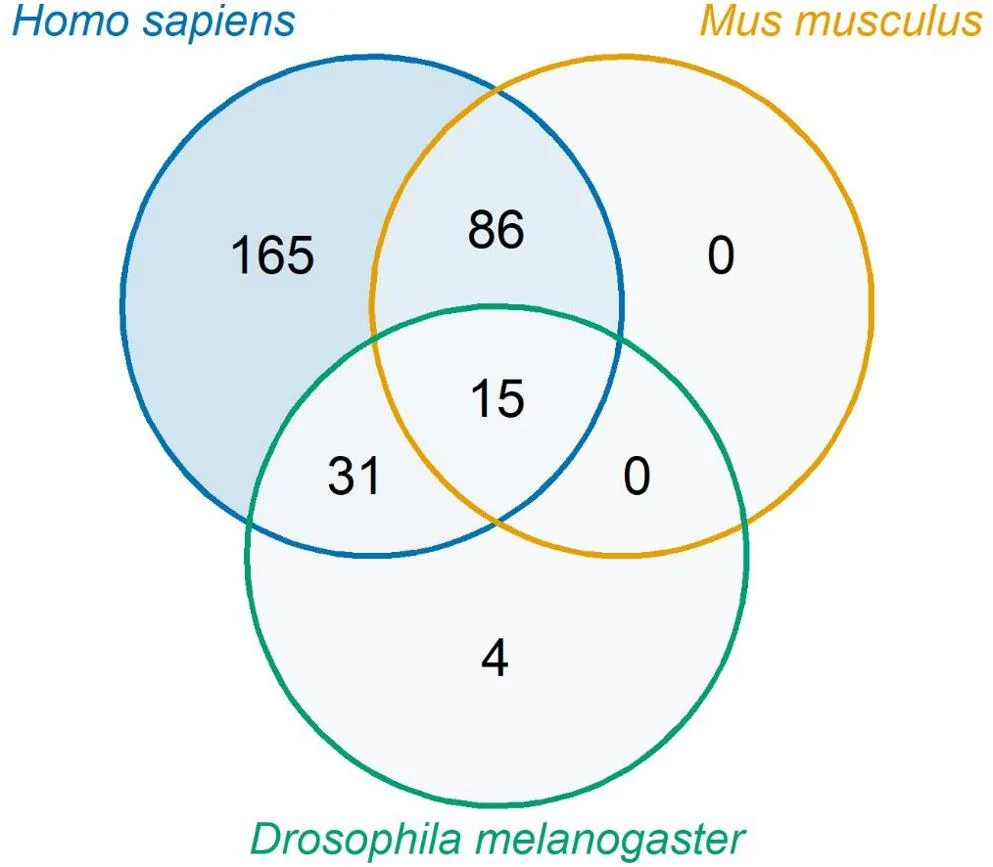
Taxonomic intersection of the core microbiome across host species. Venn diagram depicting the number of shared and unique bacterial species among *Homo sapiens*, *Mus musculus*, and *Drosophila melanogaster*. Core membership was defined by retaining species present in at least 20% of samples within each host group (prevalence threshold $\:\ge\:$ 0.2). The intersection reveals a “Universal Core” comprising 15 species shared across all three hosts, whereas mammals (*H. sapiens* and *M. musculus*) share a distinct additional core of 86 species absent in *D. melanogaster*

The intersection analysis (Fig. 10) revealed a marked asymmetry in microbiome complexity between vertebrates and invertebrates. Within the identified core, a “Universal Core” comprising 15 bacterial species (*Proteus mirabilis*, *Bacillus velezensis*,* Bacillus subtilis*,* Blautia spp.*,* Pseudomonas aeruginosa*,* Staphylococcus epidermidis*,* Enterococcus faecalis*,* Escherichia coli*,* Salmonella enterica*,* Klebsiella pneumoniae*,* Staphylococcus aureus*,* Streptococcus thermophilus*,* Lactiplantibacillus plantarum*,* Streptococcus dysgalactiae*,* Nocardia seriolae*) approximately 5% of the total detected was found to be shared simultaneously by *H. sapiens*, *M. musculus*, and *D. melanogaster*. These bacteria likely represent generalist taxa with high ecological plasticity, capable of colonizing both the simple digestive tract of an insect and the complex gut of a mammal (Douglas 2018; Wong et al. 2011). Notably, the exclusive intersection between humans and mice comprised 86 species (29%), a substantial overlap that reinforces the concept of a shared ‘mammalian microbiome’ (Ley et al. 2008; Nguyen et al. 2015) adapted to the physiology of homeothermic animals possessing complex adaptive immune systems—a trait notably absent in *Drosophila (*Hooper et al. 2012).

Regarding host-specificity and complexity, *H. sapiens* exhibited the highest unique diversity, with 165 exclusive species (55%), reflecting the structural complexity of the human colonic niche and the high individual variability associated with diet and environmental factors (David et al. 2013; Falony et al. 2016). Conversely, *Drosophila* presented an extremely reduced exclusive core consisting of only 4 species (1%) (Fig. 10). This finding indicates that most stable bacteria in the fly are not host-exclusive but are shared with other animals (forming part of the Universal Core), suggesting that the *Drosophila* microbiome functions largely as a simplified subset of environmental and generalist bacteria rather than a highly specialized community (Adair et al. 2018; Blum et al. 2013; Chandler et al. 2011).

### Abundance Dynamics within the Universal Core

**Fig. 11:**
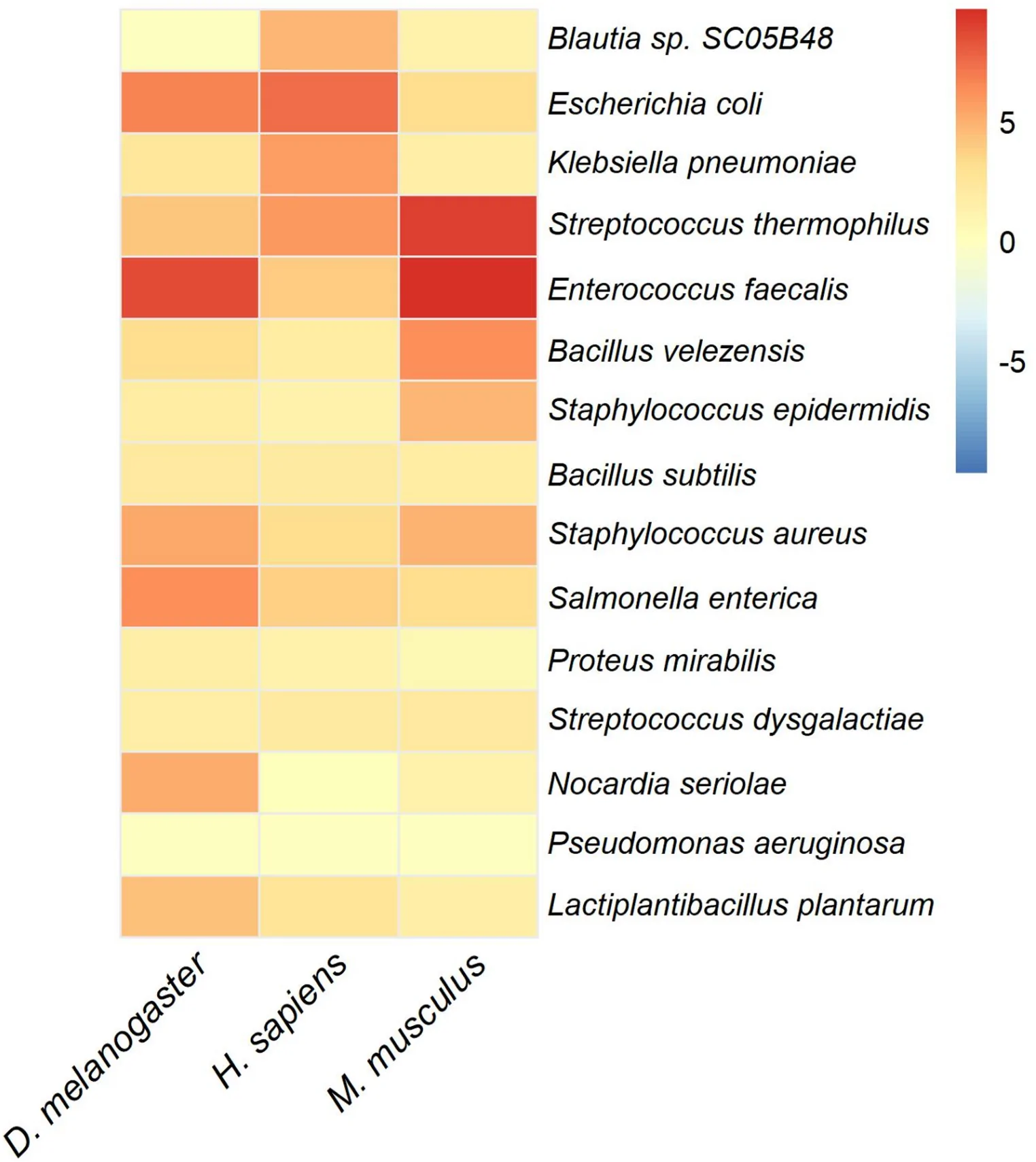
Ecological preference profiling of the microbiome Universal Core. Heatmap visualization of the 15 cosmopolitan bacterial species shared across *Homo sapiens*, *Mus musculus*, and *Drosophila melanogaster*. colour intensity represents the mean Centred Log-Ratio (CLR) abundance for each species within each host group, serving as a proxy for ecological success (Red = high relative abundance; Blue = low relative abundance). Rows are arranged via hierarchical clustering to group taxa with similar abundance distribution patterns. The analysis reveals distinct host-association signatures, distinguishing a vertebrate-enriched cluster (e.g., *Escherichia coli*, *Klebsiella pneumoniae*) from taxa with broader host plasticity, and highlighting *Lactiplantibacillus plantarum* (bottom row) as the sole core member exhibiting preferential abundance in the invertebrate host compared to mammals (Color figure online)

Although the 15 species constituting the “Universal Core” can colonize all three hosts (prevalence > 20%), their ecological success (abundance) is highly uneven. Heatmap analysis (Fig. 11) based on corrected CLR abundance averages reveals that universality of presence does not imply shared dominance. Three distinct patterns of ecological behaviour were observed.

First, a pattern of human and mammalian dominance was evident, where most universal core species exhibited a distinct preference for the vertebrate niche. Commensals and pathobionts such as *Klebsiella pneumoniae* (CLR = 4.09), *Blautia* sp. (CLR = 4.03), and *Escherichia coli* (CLR = 3.92) reached peak abundances in humans. Conversely, these taxa displayed consistently negative values in *Drosophila*, indicating that, although present in the fly, they occur at very low abundance and likely represent transients acquired from the anthropogenic environment rather than established residents.

Secondly, a shared mammalian cluster was identified. Lactic acid bacteria and Gram-positive cocci, specifically *Enterococcus faecalis* and *Staphylococcus epidermidis*, displayed positive abundances in both mice and humans. This distribution confirms their specific adaptation to the gut of homeotherms, standing in sharp contrast to their low representation in the insect host.

Finally, the analysis highlighted a unique “fly exception”. The only species within the universal core exhibiting an ecological preference for the invertebrate was *Lactiplantibacillus plantarum* (Fig. 11). This bacterium was the sole taxon with positive abundance in *Drosophila* (CLR = 0.48), significantly surpassing its abundance in humans (CLR = -0.04) and mice (CLR = -2.50). This finding identifies *L. plantarum* as the sole true symbiont within this dataset capable of thriving optimally within the acidic environment of the fly gut, distinguishing it from the transient mammalian-associated taxa.

### Global Functional Landscape of the Host-Associated Microbiome

**Fig. 12:**
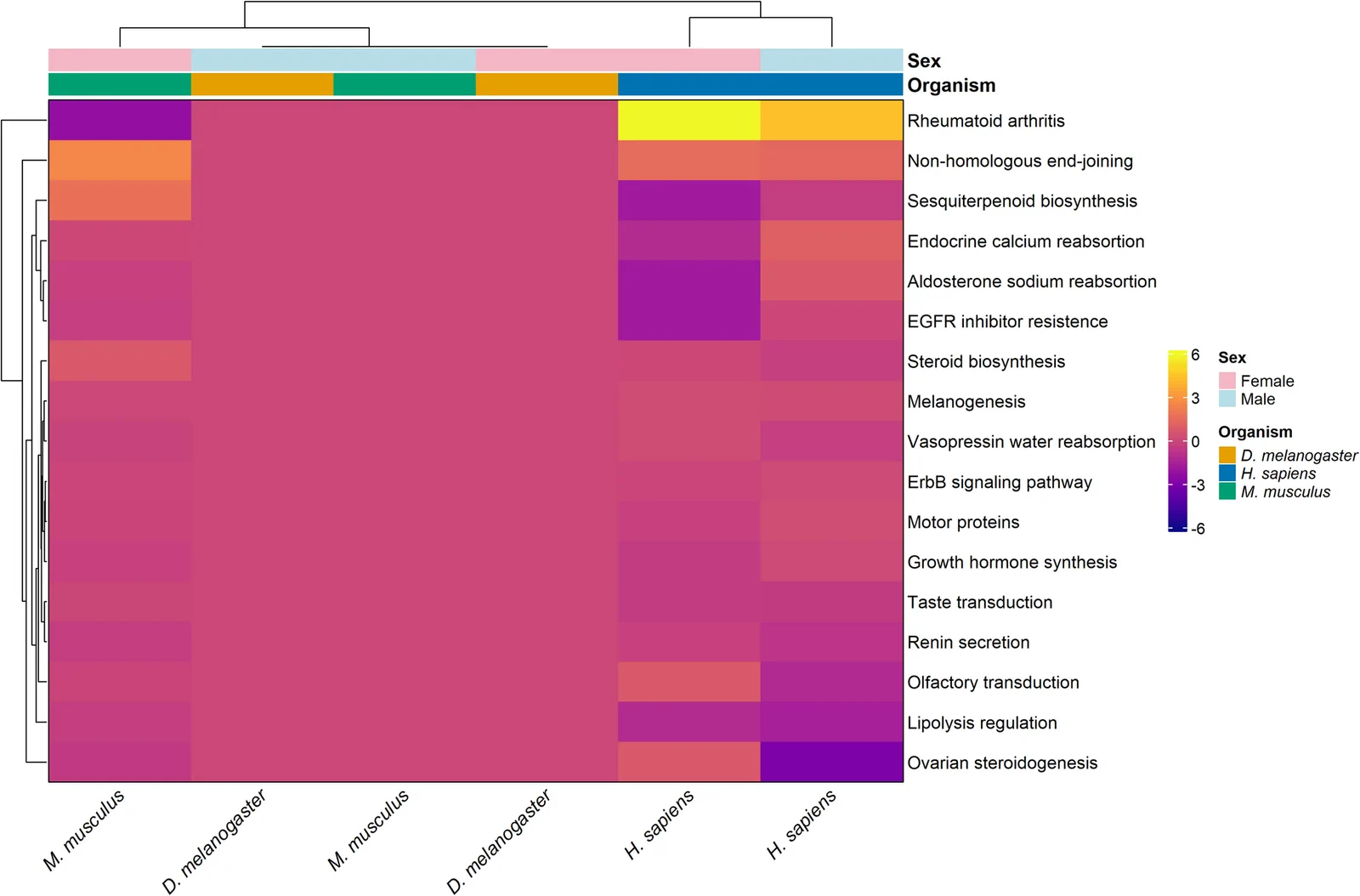
Hierarchical clustering of metabolic potential across host species. Unsupervised hierarchical clustering of KEGG metabolic pathways (rows) and host samples (columns). The heatmap visualises Centred Log-Ratio (CLR) normalised abundances, where the colour gradient ranges from blue (depletion) to red/orange (enrichment). The top annotation bar encodes host species identity (*Homo sapiens*, *Mus musculus*, *Drosophila melanogaster*). The dendrogram (top) reveals a primary stratification by host phylogeny, isolating the human samples (left clade) from the laboratory models. Notably, the clustering highlights a divergence within the murine group: whilst the female *Mus musculus* sample maintains a distinct profile, the male *Mus musculus* sample clusters within the invertebrate clade (*Drosophila*), reflecting the impact of sequencing depth on functional profile resolution (Color figure online)

To visualise the global metabolic potential across host species independent of specific pairwise contrasts, a functional heatmap based on the Centred Log-Ratio (CLR) normalised abundance of KEGG metabolic pathways was generated (Fig. 12). Unsupervised hierarchical clustering was performed to group samples based on fundamental functional similarity. The analysis revealed a primary stratification driven by both host identity and data complexity. Human samples (both male and female) formed a distinct, tightly grouped clade characterised by a widespread “functional expansion” (predominance of relative enrichment), reflecting a complex metabolic repertoire likely adapted to a varied anthropised diet and host physiology.

Notably, laboratory model organisms did not form a single unified clade. The high-depth female *Mus musculus* sample was positioned intermediate to the human cluster, whereas the low-depth male *Mus musculus* sample sequencing (depth: 53,459 reads) clustered more closely with the *Drosophila melanogaster* samples.

### Conserved Metabolic Signatures of Sexual Dimorphism

Human and murine functional profiles were integrated to delineate a “core” of sexually dimorphic microbiome functions transcending host taxonomy. By aligning the differential abundance trends of both species, a subset of metabolic pathways exhibiting concordant regulation across mammals was identified.

**Fig. 13:**
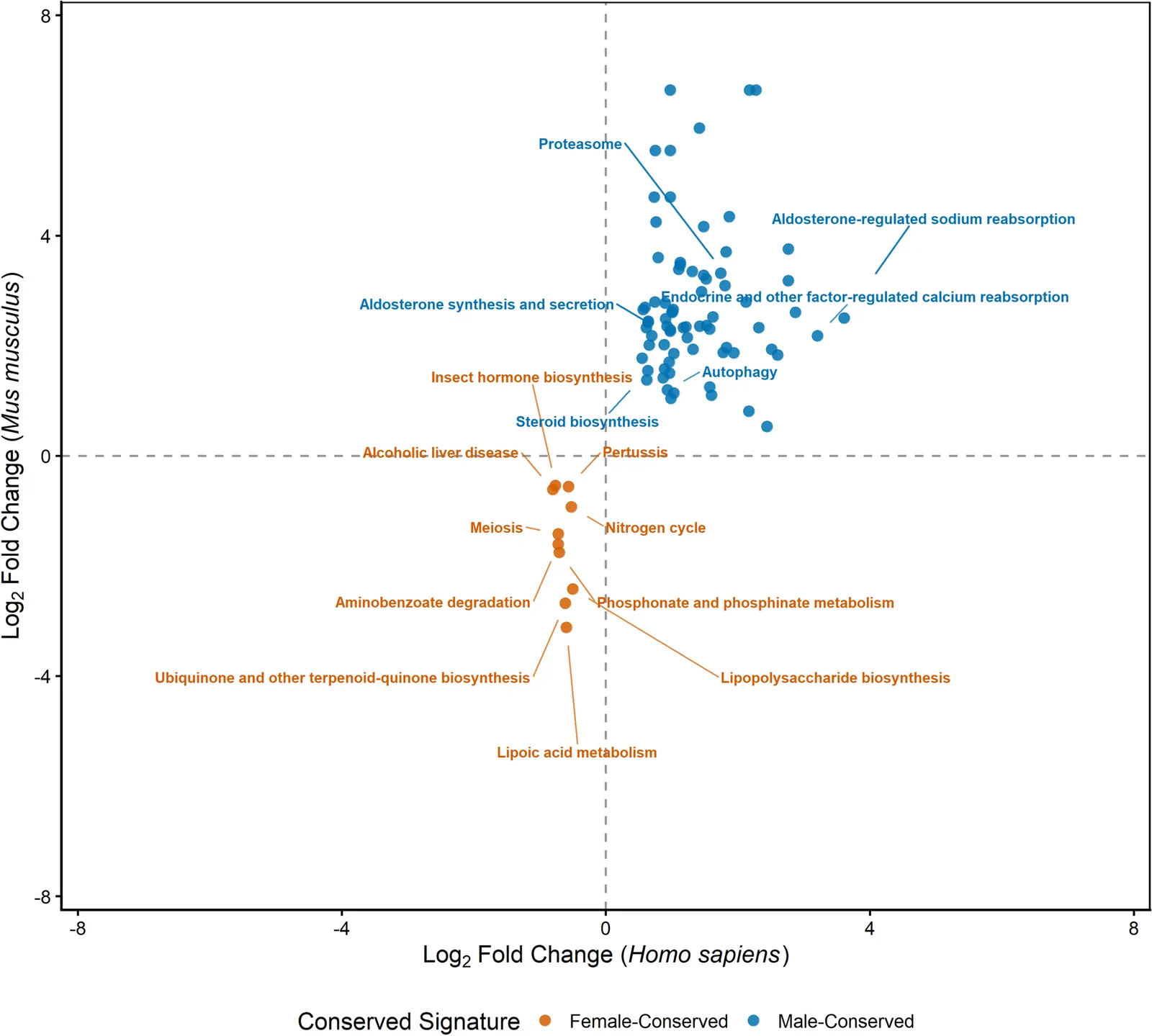
Cross-species alignment of conserved sex-specific metabolic signatures. Scatter plot illustrating the concordance of functional sexual dimorphism between *Homo sapiens* (x-axis) and *Mus musculus* (y-axis). Each point represents a KEGG metabolic pathway. The axes display the magnitude and direction of the log2 Fold Change. Note: Due to murine sample availability (*n* = 1 pooled per sex), this plot represents an algebraic trend alignment rather than a population-level statistical correlation. Pathways situated in the upper-right quadrant (Blue points) denote consistent male-bias trends, whereas those in the lower-left quadrant (Orange points) denote consistent female-bias trends (Color figure online)

Figure 13 illustrates the cross-species alignment of these functional trends. Consistent enrichment trends were observed across both species; specific pathways demonstrated a male bias (Blue), whilst a distinct subset was universally enriched in females (Orange). Importantly, despite the limitation in murine sample size, the strong directional alignment of these pathways with the high-power human dataset suggests a biological signal distinct from random noise. As detailed in Supplementary Table S2, a distinct set of conserved pathways was identified. Notably, pathways related to steroid metabolism (e.g., *Steroid biosynthesis*, *Ovarian steroidogenesis*) were consistently enriched in females, reinforcing the potential role of the microbiome in modulating host reproductive endocrinology. Conversely, the male-associated core included pathways such as Aldosterone-regulated sodium reabsorption and Endocrine calcium reabsorption, suggesting a conserved metabolic demand for these homeostatic functions within the male host environment.

## Discussion

### The Taxonomic Barrier vs. Functional Convergence

A central premise of translational microbiome research is that murine models faithfully recapitulate human host–microbe interactions (Hugenholtz and de Vos 2017 b; Nguyen et al. 2015; Walter et al. 2020). Our meta-analysis challenges the simplistic view of taxonomic equivalence whilst validating the model at a functional level. We observed substantial taxonomic divergence: the *Mus musculus* microbiome shares only a fraction of its core species with *Homo sapiens*. This finding aligns with the concept of “host filtering,” where distinct anatomical and physiological constraints—such as the murine forestomach pH and rapid transit time—select for specific microbial inhabitants adapted to each niche. The marked taxonomic divergence observed between *Homo sapiens* and *Mus musculus*, despite their shared mammalian ancestry (Brooks et al. 2016), is heavily shaped by host-specific ecological filtering at the species level (Mazel et al. 2018).

Despite this taxonomic disparity, our metabolic reconstruction reveals significant functional equivalence. This aligns with recent phylogenetic comparative approaches demonstrating that while vertebrate gut microbiota exhibits strong evolutionary conservatism, host-specific ecological niches drive substantial compositional turnover (Perez-Lamarque et al. 2023). Our data confirms that while the murine microbiome conserved the mammalian metabolic core, it possesses a distinct catalogue of genomic features compared to its human counterpart (Kieser et al. 2022). These findings substantiate a modular paradigm of functional redundancy, wherein core metabolic architectures are evolutionarily conserved despite the underlying taxonomic composition being heavily constrained by host-specific ecological filtering. Consequently, while the murine model represents a robust system for interrogating conserved microbiome-derived metabolic networks, extreme caution is warranted when extrapolating taxon-targeted therapeutic interventions directly to human clinical applications.

### Evolutionary Outgroups and Methodological Vulnerabilities

To rigorously contextualise this mammalian convergence, we incorporated *Drosophila melanogaster* as an evolutionary outgroup. The marked functional divergence observed between flies and mammals aligns with prior reports describing the reduced complexity and instability of the insect gut microbiome (Broderick and Lemaitre 2012; Douglas 2018). While our analysis identified *Lactiplantibacillus plantarum* as a dominant taxon across all hosts, this is consistent with its documented ecological plasticity and metabolic versatility rather than a specific host adaptation (Marco et al. 2009). We therefore caution against interpreting this as universal symbiosis beyond the scope of the analysis’s datasets.

Importantly, the fly microbiome exhibited a significantly reduced representation of pathways associated with complex vertebrate-associated metabolic processes. This does not imply an absence of endocrine regulation within the host but rather reflects the limited contribution of its microbiota to such functions relative to mammals as evidenced by the lack of annotated KOs related to steroid and bile acid metabolism in the *Drosophila* datasets. Consequently, whilst *Drosophila* remains invaluable for dissecting conserved innate immune mechanisms (Buchon et al. 2014), its lack of a functional mammalian metabolic repertoire limits its direct application to complex metabolic disorders. Therefore, we propose that the fruit fly should be conceptualised as an ‘evolutionarily informative but translationally constrained’ model: powerful for identifying ancient host-microbe associations, yet insufficient for modelling vertebrate-specific metabolic interfaces.

While it is difficult to make broad generalizations from a single observation, the clustering of this specific low-depth sample suggests that technical noise or insufficient sequencing depth might artificially compress the functional profile of a mammalian microbiome, making it resemble that of an invertebrate. Consequently, whilst host phylogeny is a major driver of functional architecture, technical factors influencing data complexity—specifically sequencing depth—can significantly impact global similarity patterns.

A particularly instructive finding of this study was the clustering of the low-depth murine sample with *Drosophila*, highlighting the susceptibility of functional inference to sequencing depth bias. Insufficient coverage preferentially captures abundant generalist taxa whilst underrepresenting the rare biosphere—a substantial reservoir of metabolic potential (Sogin et al. 2006). Such under sampling can artificially compress functional diversity, producing an illusion of ecological simplicity.

This observation aligns with broader concerns regarding compositional bias and depth-related artefacts in metagenomic studies (Hillmann et al. 2018; Weiss et al. 2016). Our results underscore that apparent interspecies convergence (or divergence) may reflect technical variation rather than biological reality. Adequate sequencing depth is therefore a fundamental prerequisite for capturing subdominant but functionally active taxa, mitigating the risk of erroneously dismissing valid mammalian models due to depth-induced zero-inflation artifacts.

### Conserved Sexual Dimorphism in Microbial Function

A key translational finding of this study is the identification of conserved sex-biased functional signatures across mammals. While alpha-diversity metrics revealed limited and inconsistent dimorphism, our pathway-level alignment exposed clear directional conservation between humans and mice.

Female-enriched pathways included modules associated with steroid metabolism and ovarian steroidogenesis. This is consistent with the estrobolome concept, the aggregate of microbial genes capable of modulating oestrogen metabolism via β-glucuronidase activity (Plottel and Blaser 2011). Conservation of such signatures across species suggests a co-evolved relationship between microbial metabolism and host endocrine regulation. Female-enriched pathways included modules associated with steroid metabolism and ovarian steroidogenesis. This observation is strongly supported by recent literature demonstrating that gut bacterial genes encoding steroid-transforming reductases are significantly enriched in female human microbiomes (Arp et al. 2025). This is consistent with the estrobolome concept, the aggregate of microbial genes capable of modulating oestrogen metabolism via β-glucuronidase activity (Plottel and Blaser 2011). Conservation of such signatures across species suggests a co-evolved relationship between microbial metabolism and host endocrine regulation. From an evolutionary standpoint, this conservation suggests that microbiome is actively integrated into the differing reproductive and energetic fitness strategies of male and female hosts (Jašarević et al. 2016). Female-specific enrichment of the estrobolome likely represents a co-evolved mechanism to maximise energy extraction and regulate endocrine signalling during metabolically demanding periods such as pregnancy and lactation (Koren et al. 2012).

Conversely, male-enriched pathways included modules related to sodium handling (e.g., aldosterone-regulated sodium reabsorption) and hormone-responsive metabolic regulation. Although causality cannot be inferred solely from metagenomic data, these findings are consistent with emerging evidence linking gut microbiota composition to blood pressure regulation and mineralocorticoid signalling (Wilck et al. 2017). Together, these data point towards sex as a biologically embedded dimension of microbiome function. Whilst rigorous phylogenetic analyses are required to confirm the evolutionary origin of these traits, the functional consistency observed here strongly advocates for the inclusion of both sexes in microbiome-based therapeutic studies.

### Implications for Translational Validity

Our findings suggest that the murine microbiome should not be regarded as taxonomically equivalent to human microbiome. However, selective functional conservation—particularly at the level of core metabolic processes and sex-associated signatures—supports its continued utility as a mechanistic model. Rather than asking whether the mouse is “equivalent”, more appropriate question is which functional domains are conserved and therefore legitimately translatable.

Future translational frameworks should therefore adopt a modular perspective, distinguishing conserved metabolic circuits from host-specific adaptations. Only through such functional stratification can the murine model be appropriately validated or limited in scope.

Finally, our metabolic interpretations must be contextualised within the inherent limitations and historical biases of current functional annotation databases, such as KEGG and eggNOG. These repositories are predominantly skewed towards well-characterised, culturable taxa and human-associated microorganisms, potentially underrepresenting the accessory metabolic repertoire of non-model or invertebrate microbiomes such as *Drosophila*. Consequently, a fraction of the host-specific functional divergence may be obscured by a lack of homologous annotations for niche-specific genes. Nevertheless, our conclusions regarding the functional equivalence of mammalian microbiomes rely primarily on deeply conserved, core metabolic circuits (e.g., central carbon metabolism and conserved steroid transformations), which are robustly and universally annotated across all domains of life. Thus, while database biases may limit the discovery of novel non-human pathways, they do not invalidate the robust evolutionary conservation observed in the core metabolic architecture. It must be noted that DNA-based Whole-Genome Shotgun (WGS) metagenomics captures functional potential rather than active gene expression or dynamic metabolic flux. While the stringent environmental pressures of the gut typically select against the retention of unexpressed genes, meaning genomic potential strongly correlates with functional output in these ecosystems, these DNA-based inferences remain predictive. Future multi-omics approaches, integrating metatranscriptomics and metabolomics, will be essential to definitively validate the active transcription and absolute metabolic output of the sex-specific and host-specific functional signatures identified herein.

## Conclusion

While it is well-established that the murine microbiome is not a taxonomic mirror of the human gut (Kim et al. 2024; Nguyen et al. 2015), our findings provide a quantitative map of where this divergence translates into functional shifts. We demonstrate that taxonomic divergence between *Mus musculus* and *Homo sapiens* is substantial and structurally embedded, shaped by host-specific ecological filtering and evolutionary constraints. Rather than entirely precluding translational utility, our results demonstrate that functional conservation between mammals is inherently modular. The murine microbiome preserves core metabolic engines (e.g., short-chain fatty acid production) whilst diverging substantially in specific adaptive pathways.

This provides robust metagenomic evidence supporting the Functional Redundancy Hypothesis but establishes critical boundaries; the mouse is not a miniature human ecosystem; it is a distinct biological system that converges on similar metabolic solutions only within specific functional domains. Consequently, the translational value of murine models lies not in taxonomic imitation, but in domain-specific functional conservation.

The evolutionary contrast with *Drosophila melanogaster* demonstrates that host physiological complexity is directly mirrored by microbial metabolic potential. Concurrently, our observation regarding sequencing depth highlights a potential methodological caveat: the apparent ecological simplicity in specific samples may be confounded by technical noise or insufficient depth. Resolving the rare biosphere that differentiates a mammalian gut from an invertebrate one therefore demands rigorous, deep metagenomic sequencing.

The conservation of sex-biased signatures in steroid and mineralocorticoid metabolism across species demonstrates that sexual dimorphism extends beyond a mere experimental confounder; it represents an evolutionarily conserved feature of host-microbe interactions. These findings indicate that biological sex fundamentally shapes microbiome function, establishing it as a critical variable for translational models. Ultimately, these data redefine the translational scope of the murine model. Future experimental designs must transition from assuming broad physiological equivalence to the empirical mapping of specific, conserved metabolic modules. Only through this pathway-centred validation can murine models be rigorously deployed to bridge the translational gap in human metagenomic research.

## Supplementary Information

Below is the link to the electronic supplementary material.

Supplementary Material 1. Supplementary Table S1. Metadata and sequencing metrics for the metagenomic datasets analysed in this study. The table includes public accession numbers (Sequence Read Archive, SRA), host organism identity, biological sex, experimental group, and quality control metrics for samples from *Homo sapiens*, *Mus musculus*, and *Drosophila melanogaster*.

Supplementary Material 2. Supplementary Table S2. Evolutionarily conserved sex-specific metabolic signatures across mammalian hosts. The table details the KEGG pathways exhibiting concordant differential abundance between sexes in both *Homo sapiens* and *Mus musculus*. Data includes KEGG IDs, pathway descriptions, the conserved direction of enrichment (Female-Conserved or Male-Conserved), and the specific log_2_ Fold Change magnitudes for each species.

Supplementary Material 3. Supplementary Table S3. Top functional KEGG Orthologs (KOs) enriched in Murine and *Drosophila* models. The table lists the most distinct metabolic signatures specifically enriched in the animal models when compared to the human functional baseline, ensuring symmetric representation of the inter-species comparative analysis.

Supplementary Material 4. Supplementary Figure S1. Visual assessment of batch-effect mitigation. Principal Component Analysis (PCA) plots demonstrating the clustering of samples based on Centred Log-Ratio (CLR) transformed abundance data before and after the statistical correction of technical variation. Batch effects inherent to the heterogeneous origin of the datasets were mitigated using the removeBatchEffect function from the limma package, ensuring the preservation of genuine biological variance associated with host species identity.

## Data Availability

The datasets analysed during the current study are available in the NCBI Sequence Read Archive (SRA). The complete list of accession numbers and associated metadata is provided in the Supplementary Information (Supplementary Table S1).
