# Supplementary material for: Functional Equivalence and Conserved Sexual Dimorphism in the Gut Microbiome: A Cross-Species Meta-analysis
Source: J Mol Evol. 2026 Jul 29;94(5):814–34. doi: 10.1007/s00239-026-10338-z (PMC13672462; doi:10.1007/s00239-026-10338-z)
Supplement: Supplementary file 1 [file 239_2026_10338_MOESM4_ESM.docx]

**Section 1. Bioinformatic Pipeline and Quality Control Protocols**

1.1. **Metagenomic Sequence Pre-processing and Quality Control**

Raw metagenomic reads were processed through a standardised quality control pipeline. Initial quality assessment was performed using FastQC (v0.12.1) to evaluate per-base sequence quality, adapter content, and duplication levels.

1.2. **Sequence Trimming and Filtering**

Trimming was executed using Trim Galore (v0.6.10) to remove low-quality bases (Phred score < 20) and Illumina adapter sequences. Reads shorter than 50 bp post-trimming were discarded to ensure high-quality taxonomic and functional assignments

1.3. **Computational Depletion of Host DNA**

To ensure the purity of microbial functional signals, we performed a rigorous host decontamination step. Non-microbial reads were identified by aligning raw data against the respective host reference genomes: *GRCh38.p14* for *Homo sapiens*, *GRCm39* for *Mus musculus*, and *BDGP6* for *Drosophila melanogaster*. Alignments were conducted using BWA-MEM2 with default parameters. Only unmapped reads (those not originating from the host genome) were retained for taxonomic and functional profiling.

1.4. **Sample Exclusion Criteria and Sequencing Depth Artefacts**

To account for technical variation, a stratified inclusion strategy was implemented While all samples with >1 million reads were included for broad diversity metrics (Alpha and Beta diversity), we observed that low-depth mammalian samples (e.g., murine sample SRR10905282, 0.9 GB) exhibited an aberrant functional profile, erroneously clustering with invertebrate samples in hierarchical clustering analysis. Consequently, this sample was excluded from all differential abundance analyses (Volcano Plots) and metabolic reconstructions to prevent false negatives and zero-inflation bias.

**1.5. Batch Effect Assessment and Mitigation**

Given the heterogeneous, multi-study origin of the datasets, potential technical batch effects were evaluated using Principal Component Analysis (PCA) on Centred Log-Ratio (CLR)-transformed species abundance matrices. To mitigate technical variance, the *removeBatchEffect* function from the *limma* R package was employed (Ritchie et al., 2015). The biological covariate (host organism) was incorporated into the design matrix to ensure the correction algorithm eliminated technical noise whilst preserving genuine inter-species biological signals. The efficacy of this normalisation procedure was visually confirmed (Supplementary Figure S1), demonstrating that samples clustered primarily according to their biological identity rather than their processing batch. This normalisation validates the structural integrity of the integrated dataset for subsequent downstream differential analyses.

**Supplementary Figure S1. Assessment and mitigation of technical batch effects via Principal Component Analysis (PCA).** Ordination was performed on the Centred Log-Ratio (CLR)-transformed species abundance matrix. (A) Prior to batch effect correction, the first two principal components explain 26.2% (PC1) and 17.5% (PC2) of the total variance, with samples exhibiting distinct technical clustering. (B) Following correction using the *limma* package, the axes represent 27.0% (PC1) and 18.6% (PC2) of the variance, revealing clustering driven by biological identity. Data points are coloured by processing batch (Batch 1: salmon; Batch 2: turquoise) and shaped according to host organism (Circle: *Drosophila melanogaster*; Triangle: *Homo sapiens*; Square: *Mus musculus*). Dashed ellipses indicate the 95% confidence intervals for the distribution of each batch.

**Section 2. Taxonomic Redundancy and Genomic Divergence Analysis**

**2.1. Taxonomic Saturation and Sample Size Sufficiency**

The rarefaction analysis (Supplementary Figure S2) demonstrates that for all host species, the total observed richness (taxonomic richness) reaches a clear plateau (asymptote) well before the total number of samples is reached. In the specific case of the murine dataset, taxonomic saturation is achieved at approximately 30–40 samples, as evidenced by the flattening of the accumulation curve. Beyond this point, the addition of further samples results in a negligible increase in new taxa detection. This confirms that our curated selection of 75 high-quality, untreated mouse samples provides a saturated and stable representation of the gut microbiome, capturing the ecosystem backbone necessary for the core interactome characterization without the risk of under-sampling bias. Consequently, the sample size imbalance between hosts does not compromise the validity of the cross-species comparisons, as each group has reached its respective point of taxonomic saturation.

**Supplementary Figure S2. Assessment of Sampling Adequacy via Species Accumulation Curves**

The robustness of the comparative conclusions relies on the adequate representation of microbial diversity within each host. To address sample size imbalances, we generated rarefaction curves using 999 random permutations per sampling point (Gotelli & Colwell, 2001). The results demonstrate that the murine dataset achieves taxonomic saturation at approximately 30–40 samples. The final slope of 0.1498 provides strong evidence that the current sampling effort has captured most of the resident microbiota, standardising the comparison by community completeness rather than raw sample size (Chao & Jost, 2012).

**2.2. Genomic Validation of Taxonomic Independence**

To address potential taxonomic overlap between the microbial communities of *H. sapiens*, *M. musculus*, and *D. melanogaster*, we quantified global genomic distances across 116 representative reference genomes. Given the inclusion of complex eukaryotic reference genomes (e.g. human and mouse) alongside bacterial representatives, the MinHash-based algorithm Mash v2.3 (Ondov et al., 2016) was employed for its computational efficiency and high correlation with the Average Nucleotide Identity (ANI).

The analysis demonstrates that, although certain taxa may share genus-level nomenclature across different study models, the specific strains inhabiting each host are genomically distinct according to established species-boundary criteria (ANI < 95% or Mash $D > 0.05$) (Jain et al., 2018). This divergence confirms that the co-occurrence networks identified in this study represent host-specific microbial architectures rather than artefacts of taxonomic redundancy. All visualisations were generated using the ComplexHeatmap package (Gu et al., 2016).

**Supplementary Figure S3. Heatmap of inter-host genomic divergence.**

Pairwise genomic distances (*D*) were calculated for 116 representative reference genomes using the Mash algorithm (Ondov et al., 2016). The colour scale represents the Mash distance, where values approaching 0 (blue) indicate high genomic similarity and values exceeding the 0.05 threshold (red) signify divergence at the species level (Jain et al., 2018). The top annotation bar indicates the host origin of each genome: Human (red), Mouse (blue), and *Drosophila* (green). Hierarchical clustering was performed using the Ward.D2 method. Off-diagonal regions, representing inter-host comparisons, consistently display distances above the 0.05 threshold, confirming the genomic independence of the microbial communities analysed.
